# Organic Radical-Boosted Ionic Conductivity in Redox Polymer Electrolyte for Advanced Fiber-Shaped Energy Storage Devices

**DOI:** 10.1007/s40820-025-01700-9

**Published:** 2025-03-13

**Authors:** Jeong-Gil Kim, Jaehyoung Ko, Hyung-Kyu Lim, Yerin Jo, Hayoung Yu, Min Woo Kim, Min Ji Kim, Hyeon Su Jeong, Jinwoo Lee, Yongho Joo, Nam Dong Kim

**Affiliations:** 1 Institute of Advanced Composite Materials, Korea Institute of Science and Technology, 92 Chudong-ro, Bongdong-eup, Wanju-gun, Jeollabuk-do 55324 Republic of Korea; 2 Department of Chemical and Biomolecular Engineering, Korea Advanced Institute of Science and Technology, 291 Daehak-ro, Yuseong-gu, Daejeon, 34141 Republic of Korea; 3 Division of Chemical and Bioengineering, Kangwon National University, Chuncheon, 24341 Republic of Korea; 4 Department of Materials Science and Engineering, Gwangju Institute of Science and Technology, Gwangju, 61005 Republic of Korea

**Keywords:** Redox polymer electrolyte, Hydroxy-TEMPO, Ionic conductivity, Self-exchange reaction, Fiber-shaped energy storage devices

## Abstract

Fiber-shaped energy storage devices (FSESDs) with exceptional flexibility for wearable power sources should be applied with solid electrolytes over liquid electrolytes due to short circuits and leakage issue during deformation. Among the solid options, polymer electrolytes are particularly preferred due to their robustness and flexibility, although their low ionic conductivity remains a significant challenge. Here, we present a redox polymer electrolyte (HT_RPE) with 4-hydroxy-2,2,6,6-tetramethylpiperidine-1-oxyl (HT) as a multi-functional additive. HT acts as a plasticizer that transforms the glassy state into the rubbery state for improved chain mobility and provides distinctive ion conduction pathway by the self-exchange reaction between radical and oxidized species. These synergetic effects lead to high ionic conductivity (73.5 mS cm^−1^) based on a lower activation energy of 0.13 eV than other redox additives. Moreover, HT_RPE with a pseudocapacitive characteristic by HT enables an outstanding electrochemical performance of the symmetric FSESDs using carbon-based fiber electrodes (energy density of 25.4 W h kg^−1^ at a power density of 25,000 W kg^−1^) without typical active materials, along with excellent stability (capacitance retention of 91.2% after 8,000 bending cycles). This work highlights a versatile HT_RPE that utilizes the unique functionality of HT for both the high ionic conductivity and improved energy storage capability, providing a promising pathway for next-generation flexible energy storage devices.

**Supplementary Information:**

The online version contains supplementary material available at 10.1007/s40820-025-01700-9.

## Highlights

- Developed a versatile HT-based redox polymer electrolyte with exceptional ionic conductivity (73.5 mS cm^−1^) through self-exchange reaction-based ion hopping mechanism and enhanced polymer chain mobility.
- Achieved superior electrochemical performance (25.4 Wh kg^−1^ at 25,000 W kg^−1^) in fiber-shaped energy storage devices without additional active materials by utilizing HT as both ionic conductor and redox-active species.

**Supplementary Information:**

The online version contains supplementary material available at 10.1007/s40820-025-01700-9.

## Graphical abstract

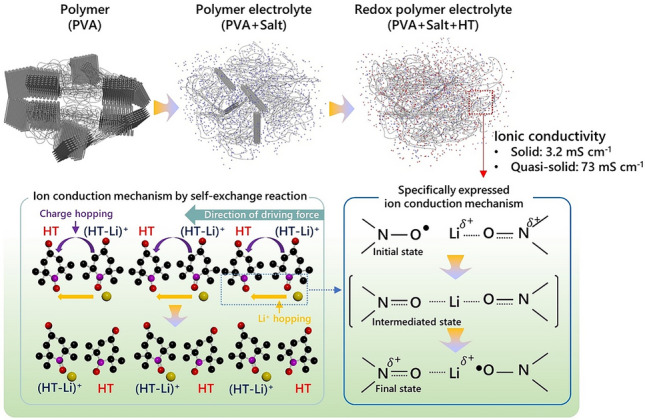

## Introduction

As the global market for state-of-the-art flexible electronics has grown over the past few years, fiber-shaped energy storage devices (FSESDs) that feature an exceptional flexibility and/or compatibility with textiles/fabrics have become a peerless class in the field of wearable power sources [1–6]. The electrolyte, which is one of the components for FSESDs, should preferably be a solid type rather than a liquid type to inhibit short circuits and electrolytes leakage issues during deformation in wearable application [6]. Solid electrolytes can be classified into three main materials: sulfides, oxides, and polymers [7]. Among these, polymer electrolytes are particularly suitable for loading FSESDs due to their strong adhesive properties, outstanding physical flexibility and stability [8, 9]. However, enhancing ionic conductivity remains a significant challenge for polymer electrolytes in FSESDs applications.

One of the innovative insights for high ionic conductivity of polymer electrolytes is to integrate redox additives into the polymer matrix [10–13]. It has been known that the additives can promote the ionic conduction by transforming a semicrystalline phase of the matrix (i.e., polyvinyl alcohol, PVA) into an amorphous phase [10, 14, 15]. Importantly, by integrating redox materials into the matrix to replace the pseudocapacitive materials in the composite fiber electrode, they can minimize the interfacial complexity that the fiber electrode often encounters. The most widely studied redox additives are inorganic species such as halogens (Br, I) or metal ions (Cu, Fe, etc.). However, these are prone to side reactions, such as water decomposition or the inverse growth of the additives on the electrode surface [16]. Another example of such redox additive is that of small molecular hydroquinone, which displays very high redox reversibility and thus the electrochemical performance [15, 17, 18]. Despite the initial success, low solubility of hydroquinone in an aqueous electrolyte and activation only in acidic environments has been notable limitations [12, 15, 19, 20]. It is thus highly desirable that one develops a redox-based additive platform including higher hydrophilicity and operation in a wide range of chemical environment.

Stable open-shell nitroxides incorporating 2,2,6,6-tetramethylpiperidine 1-oxyl (TEMPO) moiety have attracted recent attention in both solid-state electronics and electrolyte-based systems owing to a number of combinations with functional groups and commercial availability [21–24]. Among these, 4-hydroxy-TEMPO (HT) features its additional hydrophilicity in addition to fast redox capability of the nitroxides [25–27]. Specifically, an electron transfer rate constant of HT is approximately in an order of 10^–2^ cm s^−1^, which is several orders of magnitude higher than that of other redox additives [28]. Our previous study showed that a molten state of HT exhibits an exceptional solid-state conductivity by hopping process [28]. Moreover, there have been reports that the solid-state conductivity of HT can be significantly improved through ionic doping with other metal ions, such as lithium [29]. While several fundamental studies have indicated that HT is a promising candidate for enhancing ionic conductivity and facilitating fast redox reactions, its incorporation into a polymer matrix as a solid-state electrolyte has not yet been extensively studied.

Herein, we propose a versatile HT-based redox polymer electrolyte (HT_RPE) tailored for FSESDs characterized by its high ionic conductivity. It has been observed that the radicals of HT, active redox species, are well-preserved in a composite with lithium ions and the polymer backbone. The semicrystalline nature of polymer backbone becomes more amorphous after adding HT in the HT_RPE. Owing to hopping mechanism facilitated by self-exchange reactions of HT in this environment, our developed HT_RPE displayed a high ionic conductivity of 73.5 mS cm^−1^. FSESDs with the applied HT_RPE exhibit an outstanding electrochemical performance (energy density of 25.4 Wh kg^−1^ at a power density of 25,000 W kg^−1^) without active materials, with its excellent mechanical durability (capacitance retention of 91.2% after 8000 bending cycles) compared to previous studies. Our study highlights an attractive strategy to develop a versatile HT_RPE that can be applied in energy storage devices of various form factors.

## Experimental Section

### Materials

The graphene oxide (GO) dispersion (V-51, 1 wt%) was obtained from Standard Graphene Inc. Single-walled carbon nanotubes (TUBALL) were sourced from OCSiAl. The lithium perchlorate (LiClO_4_, 95%) was procured from Samchun. Additional chemicals, including acetone (99.5%), chlorosulfonic acid (CSA, 99%), and PVA (M.W.: 89,000–98,000, 99 + %, hydrolyzed), were purchased from Sigma-Aldrich. HT (free radical, 98 + %) was purchased from Thermo Scientific Chemicals.

### Fabrication of the HT_RPEs

The HT_RPEs utilized in this study were prepared by a simple mixing method. Specifically, an aqueous solution of 1 M LiClO_4_ was prepared, to which was added a designated molarity of HT. The resultant mixtures were then stirred for 30 min at room temperature. Five different sets of HT_RPEs were prepared, with variable amounts of HT. In detail, 0, 0.1, 0.5, 1.0, and 2.0 M of HT were added to 1 M LiClO_4_, respectively (these are referred to as **T-0**, **T-1**, **T-5**, **T-10**, and **T-20**, respectively, in the main text). To each mixture was added 1 g of PVA, which was then stirred vigorously at 85 °C for 20 min. All the HT_RPEs fabricated displayed optical clarity with the characteristic orange color.

### Production of the Liquid Crystal-Spun Carbon Nanotube Fiber

Chlorosulfonic acid (CSA) was employed as a thermodynamic solvent to develop the lyotropic liquid crystalline (LC) phase of carbon nanotubes (CNTs). This approach enabled the preparation of highly concentrated CNT dispersions (30 mg mL^−1^) while preventing significant aggregation, making them suitable for wet-spinning. The prepared CNT solution was loaded into an injection glass syringe and then extruded into an acetone coagulation bath at 0.05 mL min^−1^. The resulting fibers were continuously collected on a winder operating at 3 m min^−1^. Finally, the collected CNT fibers (CNTFs) underwent acetone washing and were dried in a vacuum oven at 170 °C overnight.

### Preparation of Vertically Deposited Graphene Oxide on the CNTFs

The vertically deposited graphene oxide on the CNTFs (VG@CNTFs) with 3D structure was prepared via a modification of an electrophoretic deposition process reported previously [4]. VG@CNTFs were prepared by electrophoretic deposition using an aqueous electrolyte containing 0.7 mg mL^−1^ of graphene oxide (GO) and 0.5 M of LiClO_4_. The CNTF, Pt foil, and Ag/AgCl (saturated 3 M KCl) were used as a working, counter, and a reference electrode, respectively. Then, a constant voltage of − 1.3 V was applied for 20 s in a CHI 920D electrochemical workstation. The resultant product of the deposition process was freeze dried immediately to preserve the 3D porous structure of the VG@CNTF.

### Fabrication of Symmetrical FSESDs with HT_RPEs

The fabrication of the symmetrical FSESDs was carried out by taking VG@CNTF, without typical pseudocapacitive materials, as both positive and negative electrodes. The conventional polymer electrolyte and HT_RPEs were employed as quasi-solid-state electrolytes, which served as a separator in the FSESDs configuration. The assembly process begins with pre-coating of the quasi-solid-state electrolyte on individual VG@CNTFs (length: 5.5 cm). Subsequently, both electrolyte-coated VG@CNTFs are intertwined to form a twisted structure. An additional layer of quasi-solid-state electrolyte is applied to the assembled VG@CNTF//VG@CNTF cell. The resultant FSESDs were encapsulated by urethane for protection.

### Characterization

The surface morphology of the fiber electrode and FSESDs were monitored by field-emission scanning electron microscopy (SEM, FEI NanoSEM 450 NOVA, 10 kV, FEI). The XRD patterns to identify the amorphous nature of conventional polymer electrolyte and HT_RPE were recorded by an X-ray diffractometer (XRD, Rigaku SmartLab, Cu Kα radiation, 2θ scanning range of 5°–70°). The chemical state of the samples was analyzed by X-ray photoelectron spectroscopy (XPS, Thermo Scientific K-Alpha, Thermo Fisher Scientific). The presence of the nitroxides was verified by the ultraviolet–visible spectroscopy (UV–vis, V-670). The glass transition temperature was measured by Differential scanning calorimetry (DSC, Discovery DSC 2500, TA Instruments). The ionic conductivities of the system for polymer electrolytes were calculated from the EIS (CHI 920D) with an amplitude of 10 mV at a frequency range of 0.1–10^6^ Hz. The ionic conductivity using the 4-probe electrodes with working electrode, working sensor, reference electrode and counter electrode composed of gold wire in the in-plane system is calculated by following equation:

1 $$\sigma =\frac{L}{{R}_{\text{bulk}}S}$$

where $\sigma$ is the ionic conductivity, *L* is the distance between working sensor and reference electrode, *R* is the bulk resistance of the polymer electrolyte, and *S* is the cross-sectional area of the polymer electrolyte. The activation energy calculated from the temperature-dependent variation of ionic conductivity was evaluated by the Arrhenius equation, expressed as:

2 $$\sigma ={\sigma }_{o}\text{exp}\left[\frac{{E}_{\text{a}}}{\text{kT}}\right]$$

where ${\sigma }_{o}$ is the pre-exponential factor, ${E}_{\text{a}}$ is the activation energy, *k* is the Boltzmann constant, and *T* is the absolute temperature. The Li^+^ transport number was determined using combined chronoamperometry test and AC impedance measurements. This method employs a symmetric Li/electrolyte/Li cell configuration in a coin cell format. Using equation:

3 $${t}_{{\text{Li}}^{+}}=\frac{{I}_{s}{(\Delta V-R}_{0}{I}_{0})}{{I}_{0}{(\Delta V-R}_{s}{I}_{s})}$$

where Δ*V* represents the applied potential of 20 mV and *I*_0_ and *I*_s_ are the initial and steady-state currents from DC polarization, respectively. *R*_0_ and *R*_s_ are the initial and steady-state interfacial resistances from electrochemical impedance spectroscopy, respectively.

### Electrochemical Measurements

The electrochemical performance and characteristics were studied on a CHI 920D electrochemical workstation. The electrochemical analysis to identify the optimal HT concentration was performed by cyclic voltammetry (CV) at a scan rate of 0.1 V s^−1^ in a three-electrode system. The working, counter and reference electrodes were VG@CNTF, Ag/AgCl (3 M KCl), and Pt mesh, respectively. The electrochemical performance and characteristics of FSESDs were measured by CV (scan rates: 10–1,000 mV s^−1^), the galvanostatic charge–discharge (GCD, current densities: 50–200 A g^−1^), and electrochemical impedance spectroscopy (EIS, frequency: 0.1–1 MHz, the amplitude of 0.01 V). The specific capacitance of FSESDs was evaluated from the GCD curves at different current densities by the following equation:

4 $$C_{SP}(F g^{-1})= \frac{I\times \Delta t}{m\times \Delta V}$$

where *C*_SP_ is the gravimetric specific capacitance, *I* is the discharge current (*A*), $\Delta t$ is the discharge time (*s*) without the IR drop, and m is the total mass of the fiber electrode (*g*), and $\Delta V$ is the voltage window except for the IR drop. The energy and power densities were calculated by the following equations:

5 $$E=\frac{1}{2}{C}_{\text{SP}}\times {(\Delta V)}^{2}$$

6 $$P=\frac{E}{\Delta t}$$

where *E* is the energy density (Wh kg^−1^) and *P* is the power density (W kg^−1^). The electrochemical performance under heating up to 85 °C was evaluated with CV. To validate the FSESDs’ potential as a wearable power source, CV was measured under various physical deformations including bending, twisting, and crumpling. To identify the overall stability, the FSESDs were charged/discharged 10,000 times at a current density of 50 A g^−1^ and 8,000 times of bending cycles, with the bending angle from 0° to 180°.

### Computational Methods

Density functional theory (DFT) calculations were performed using the Gaussian software package [30]. The geometry optimizations and binding energies were obtained under the B3LYP exchange–correlation functional with the 6–311 +  + G** basis set. The binding energy between molecules was calculated as the difference between the energy of the complex and the sum of the energies of the isolated reactant molecules. All structures were fully optimized without any geometric constraints.

## Results and Discussion

### Design HT_RPEs Considering Redox Activity of HT

**Fig. 1:**
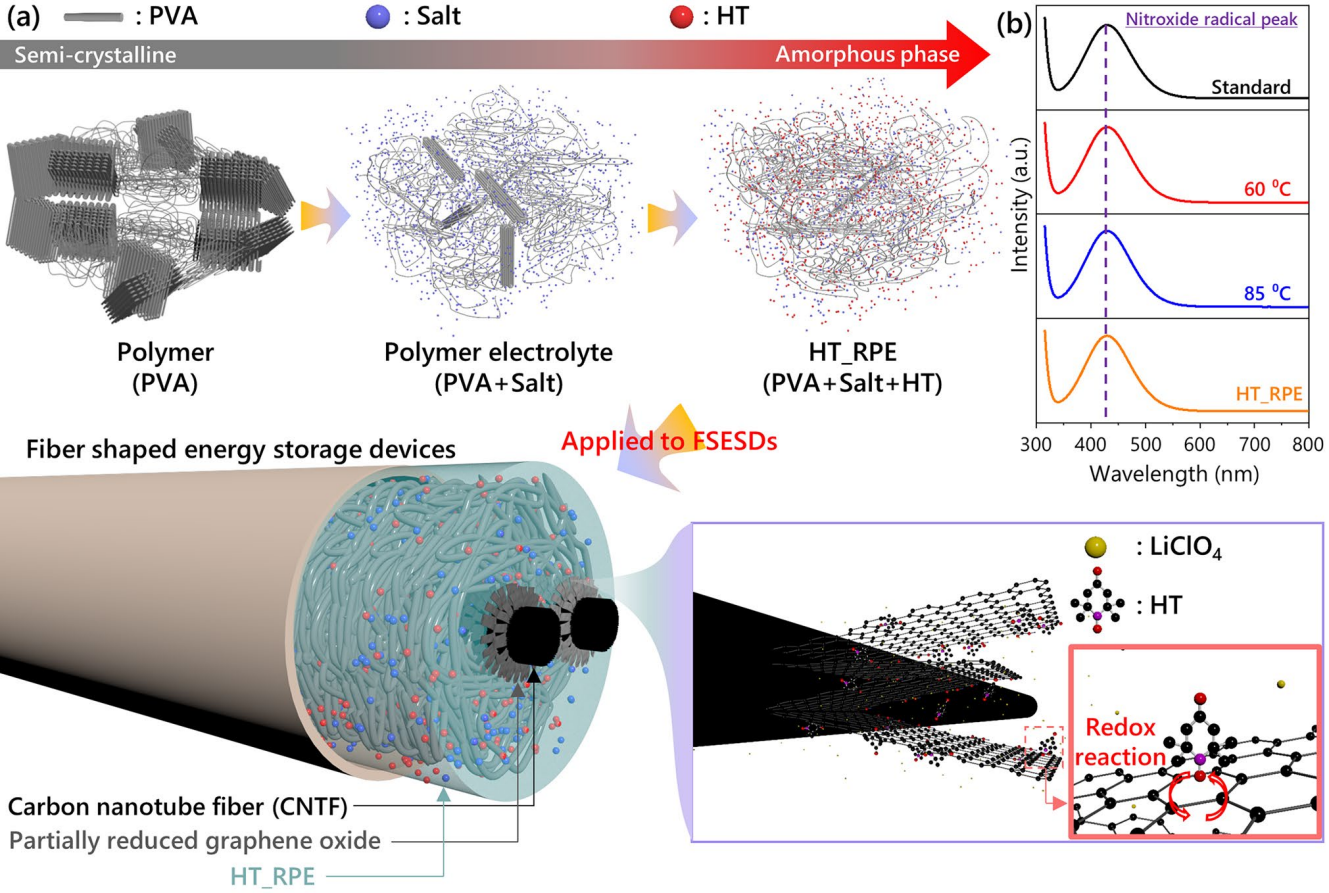
**a** Schematic illustration on the fabrication process of FSESD with for HT_RPE as an electrolyte. **b** UV–Vis profiles of the open-shell sites during the device fabrication

A schematic illustration demonstrates our design concept for HT_RPE, which is applicable for solid-state symmetric FSESDs (Fig. 1a). Prior to the fabrication of the HT_RPE, various combinations of HT and a salt (KOH, H_2_SO_4_, LiClO_4_, and NaCl) that is commonly adopted in a supercapacitors were explored in the three-electrode configuration using VG@CNTFs as the working electrode, Ag/AgCl as the reference electrode, and Pt mesh as the counter electrode. First, the electrolyte with HT alone displayed no redox reaction and only a resistive behavior in the CV profile (Fig. S1). Also, this electrolyte showed the high charge transfer resistance and diffusion resistance in electrochemical impedance spectroscopy. The HT electrolyte without supporting electrolytes exhibited insufficient ionic conductivity for an effective cell performance. In stark contrast, the HT-based electrolyte with diverse supporting electrolytes displayed effective redox reactions of HT at all pH regimes (Fig. S2). The redox reaction of HT is rarely observed in the alkaline condition with KOH. For the acidic condition with H_2_SO_4_, redox reaction of HT is observed, although redox activity derived from HT shows a low value. Interestingly, the faradaic reaction of HT is clearly confirmed in the weak acidic condition with LiClO_4_ and neutral condition with NaCl. In particular, the redox activity of HT with LiClO_4_ was the most prominent among different supporting electrolytes, based on an increased capacitance on the CV experiments (Fig. S3).

Based on the results, we fabricated the HT_RPEs incorporating PVA as a matrix, LiClO_4_ as a salt, and HT as a redox additive (In the below text, T-X refers to the 0.X molar concentration of HT in the HT_RPEs). The resultant HT_RPEs displayed optical clarity, indicating the non-aggregating nature of the components even at high concentrations (Fig. S4). The active open-shell sites in HT could be detected by the presence of a prominent peak at 420 nm in UV–Vis spectroscopy (Fig. 1b) [28]. During the preparation of the HT_RPE, the peak associated with HT maintains its position without any spectral shift. It indicates that the active radicals were unaffected at all stages of the device fabrication. Typically, the radicals of the HT are effective redox sites in the electrochemical reaction, which means that electron transfer can occur between the carbon electrode and HT in the HT_RPE. In particular, L. Guan et al. have reported that defective carbon materials such as reduced graphene oxide (r-GO) offer strong adsorption energy toward HT [31]. Thus, it is anticipated that electrochemical activity of FSESDs with HT_RPE and VG@CNTF is further enhanced by interaction between HT and VG.

### Mechanistic Understanding of Structure–Property Relationships in HT_RPEs

**Fig. 2:**
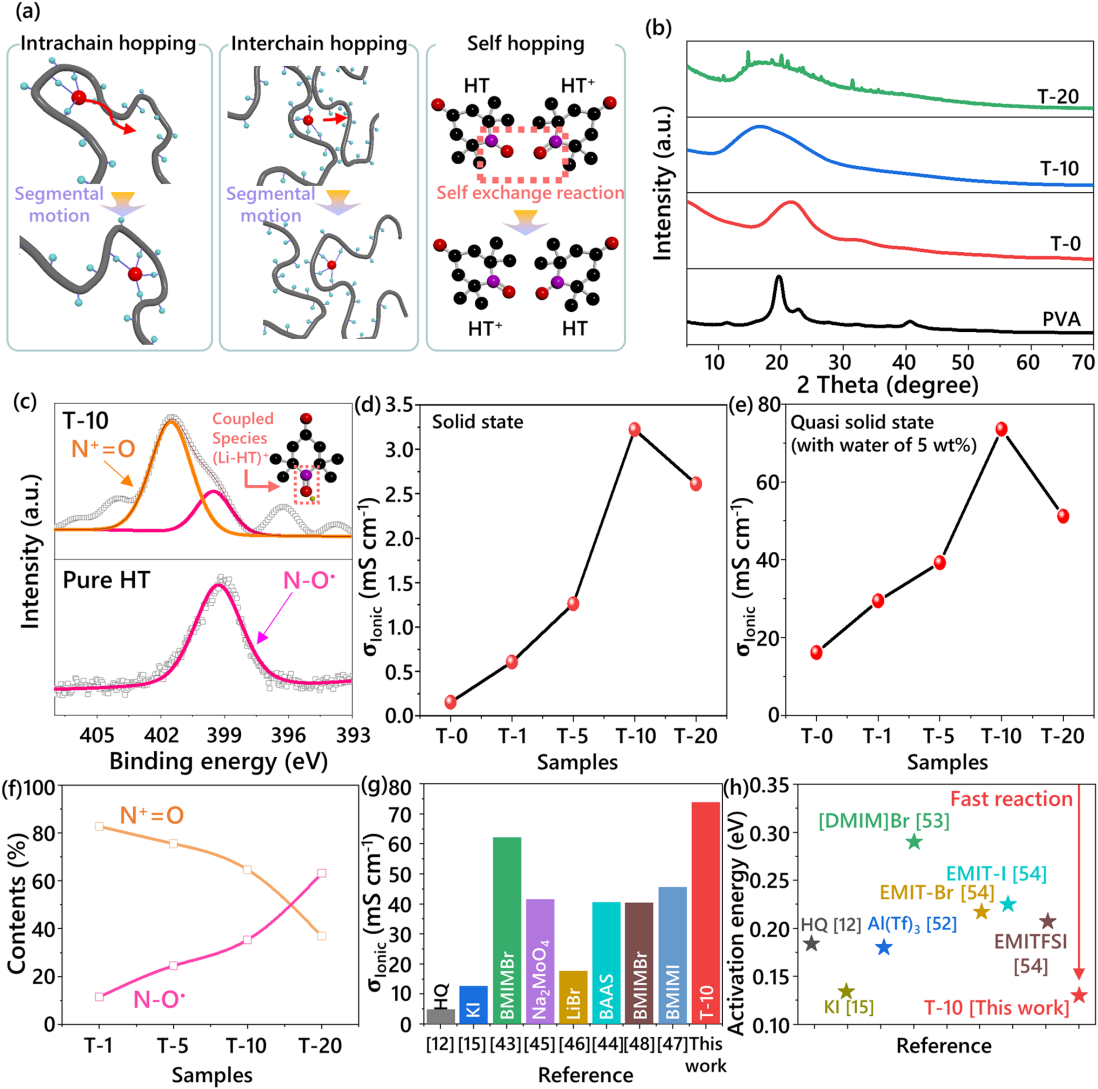
**a** Schematic illustration of the charge transport mechanism in the HT_RPEs. **b** Variation of XRD patterns. **c** High-resolution XPS spectra (Bottom: pure HT, Top: **T-10**, inset. schematic illustration of coupled species by Li^+^ ion). Comparison on the ionic conductivities with respect to the amount of HT in the HT_RPEs at 298.15 K **d** solid-state polymer electrolytes, **e** quasi-solid-state polymer electrolytes. **f** The change of chemical species in the tested RPE series. **g** The comparison of ionic conductivity in diverse redox polymer electrolyte. **h** Activation energies calculated from Arrhenius plots of temperature-dependent ionic conductivity

Generally, charge transport in polymer electrolyte with rubbery state follows the concept of intra- and interchain hopping mechanism assisted by segmental motions of polymer chains that comprise the matrix (Fig. 2a, left and middle panel) [32, 33]. Here, a charge bearing species such as LiClO_4_ and HT first coordinates with polar groups on polymer chains and then jumps to adjacent coordination sites assisted by free volume generated via segmental motions of the chains. The transport can either be in an intra- or in an interchain fashion and is closely related to the chain mobility of the constituent matrix. Specifically, the degree of crystallinity of the polymer plays an important role in determining the overall performance of the polymer electrolytes, as the amorphous nature of the polymer tends to facilitate the ion transport owing to the endowed better flexibility [34]. Figure 2b displays XRD spectra of the HT_RPE components with the different compositions for comparison. The XRD pattern of the pristine PVA displays a notable semicrystalline structure with (101) and (200) planes. Typically, the crystallinity of PVA decreases in PVA-LiClO_4_ systems (**T-0**), which is attributed to the dissolution of salt into the polymer matrix. Interestingly, even in the HT_RPE system from **T-1** to **T-10**, principal diffraction pattern associated with the pristine HT is absent (Figs. S5 and S6). This phenomenon indicates that the redox additive has also been completely dissolved in the polymer matrix owing to the high solubility of HT. However, **T-20** shows the characteristic peaks related to the HT, suggesting that excessive amounts of HT cannot be fully accommodated within polymer matrix. With increasing molar concentration of HT, the amorphous nature of HT_RPE is maximized with broader characteristic peaks, indicating the role of HT as a plasticizer in transforming crystalline domain into amorphous region that can further facilitate the charge transport and the ionic conduction of the HT_RPE system. The change in glass transition temperature (*T*_g_) with increasing concentration of HT was measured by DSC (Fig. S7). The DSC profile of T-0 system, containing only salt in PVA, displays a prominent peak at 356 K. In contrast, the incorporation of a small amount of HT (**T-1**) results in a dramatic reduction in the enthalpy and *T*_g._ The incorporation of HT into the polymer matrix increases the interchain spacing, resulting in enhanced chain mobility at lower temperature. Furthermore, HT_RPEs with higher HT content (≥ **T-5**) show complete disappearance of the *T*_g_. This phenomenon can be attributed to the larger molecular dimensions of HT, which enable penetration into the interstitial spaces between polymer chains, leading to enhanced free volume in the amorphous regions. As a result, polymer electrolyte systems **T-5**, **T-10**, and **T-20** became more rubber-like characteristics, enabling ion transport channels via intra- and interchain hopping mechanisms by segmental motion of polymer chains at room temperature.

### Enhancement of Ionic Conductivity by Chemical Interactions

XPS was utilized to elucidate interaction between radicals and LiClO_4_ in the HT_RPE system. Typically, pure HT is characterized by a singlet binding energy at 399.3 eV in a high-resolution N_1s_ spectrum, due to the dominance of the nitroxide species (N−O^•^ and/or HT^•^) in the absence of any dopants (Fig. 2c, top panel) [35]. In contrast, the addition of the salt resulted in the systematic generation of oxoammonium species (N^+^=O and/or HT^+^), which was identified at 401.8 eV as a shoulder (Fig. 2c, bottom panel and Fig. S8) [35]. The occurrence of N^+^=O of the HT_RPE series is attributed to the formation of the coupled species (Li-HT)^+^, as reported previously in our study, where the origin of the coupling is thought to be driven by the reducing nature of Li and the oxidizing nature of HT [29]. Importantly, the presence of such ionic coupling is expected to result in an overall increase in the ionic conductivity of the HT_RPEs following the generation of an additional ionic pathway [29]. Indeed, all the tested HT_RPEs showed a significant increase in the ionic conductivity compared with **T-0** (Fig. 2d, e). To precisely comprehend the origin of increased ionic conductivity, electrochemical impedance characteristics of T-0 and HT_RPEs were measured in their solid state. Despite adding a small amount of HT into PVA-based polymer electrolytes, the ionic conductivity of **T-1** significantly increases compared to **T-0**, which can be attributed to both the plasticizing effect of HT and the redox shuttling mechanism facilitated by N=O^+^ groups. In the case of **T-5**, the further enhancement in ionic conductivity can be attributed to the increased amorphous region and the glass-to-rubber transition by HT additive. Impressively, **T-10** reaches an outstandingly high ionic conductivity of 3.2 mS cm^−1^ due to ion transport by radical hopping process (see detailed discussion below). It should be noted, however, that the ionic conductivity trend showed its maxima at **T-10**, where further addition of HT resulted in a decrease in the value. Considering that quasi-solid-state electrolytes can provide both high ionic conductivity and mechanical stability, HT_RPEs with 5 wt% water content are further investigated (Fig. 2e). Water serves as a potent plasticizer, transforming the majority of the polymer matrix into amorphous regions and maintaining the rubbery state (Fig. S9). Therefore, the quasi-solid-state **T-0** electrolyte exhibits substantially enhanced ionic conductivity compared to its solid-state counterpart, attributed to the dual functionality of water as a plasticizer expanding the rubbery phase and facilitating ionic transport via the vehicle mechanism. In quasi-solid-state HT_RPEs, the role of water becomes more sophisticated. Density functional theory (DFT) calculations demonstrate that the binding energy between Li^+^ and HT (− 2.26 eV) is significantly higher than that of Li^+^ and H_2_O (− 1.59 eV), indicating that Li^+^ maintains its preferential coordination with HT rather than forming water complexes (Fig. S10). Instead of participating in Li^+^ transport directly, water transforms the environment of HT_RPEs, enabling molten state-like mobility of HT. This enhanced mobility of HT in quasi-solid-state RPEs promotes improved (HT-Li)^+^ contacts, thereby amplifying self-exchange reactions. The **T-1** shows twofold increased ionic conductivity than **T-0** due to redox shuttling mechanism by occurrence of N^+^﻿=O groups. The improved ionic conductivity of **T-5** is suggested by the increased absolute amount of HT. Remarkably, the ionic conductivity reaches its maximum value of 72 mS cm^−1^ with **T-10**, demonstrating the synergistic effect of increased HT content and enhanced radical hopping processes in the quasi-solid state. However, as observed in T-20, excessive HT content results in aggregation of free ions, leading to a decrease in ionic conductivity despite the presence of water molecules.

### Understanding the Origin of Enhanced Ionic Conductivity in HT_RPEs

To further elucidate the origin of these behaviors, we analyze the relative content of each N–O species based on the deconvolution results from high-resolution XPS spectra (Fig. S8). Specifically, the fraction of N−O^•^ keeps increasing from 18% to 64% from **T-1** to **T-20** samples, while that of the N^+^=O decreases from 82% to 36% (Fig. 2f). The ratio between these species was further verified by Raman spectroscopy, which is considered a non-invasive characterization technique (Fig. S11). While the **T-0** exhibits the baseline spectrum, HT_RPEs show separated peaks corresponding to N−O^•^ at 1393 cm^−1^ and N^+^=O at 1405 cm^−1^. In agreement with the XPS analysis, Raman spectroscopy demonstrates similar trends in the relative intensities of N−O^•^ and N^+^=O peaks across various HT concentrations. From these results, it can be hypothesized that the quantitative relationship between the HT^•^ and HT^+^ affects ionic conductivity [36]. Furthermore, this quantitative relationship may imply a correlation with the distance between the both species, which may be important in polymer electrolytes system where intermolecular distances are sufficiently close to interact with each other. In the environment where the radical molecules are highly densified, HT is capable of very fast and homogeneous intermolecular charge transfer by hopping through redox self-exchange reaction [28]. The self-exchange reaction can simply be described as (Fig. 2a, right panel):

7 $${\text{HT}}^{.}+{\text{HT}}^{+}\rightleftharpoons {\text{HT}}^{+}+{\text{HT}}^{.}$$

**Fig. 3:**
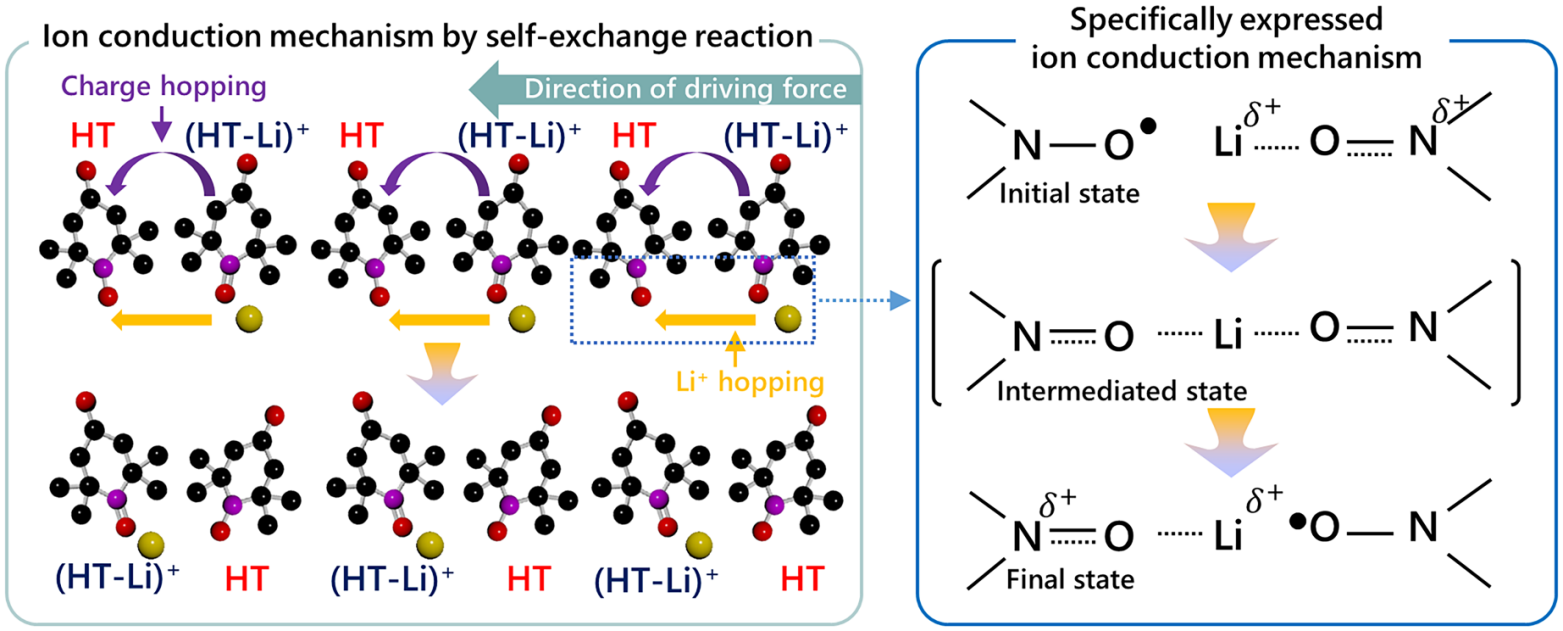
Schematic illustration of the ion conduction mechanism in HT_RPE systems

The charge transfer in this regime is characterized by shallow energy barriers, which occurs through the migration of electron without alteration of bonding [37]. In a concentrated electrolyte, Marcus-Hush theory for explaining the variation of self-exchange rate constant according to the hopping distance between HT and ${\text{HT}}^{+}$ is described as:

8 $${k}_{\text{hop}}={\left(\frac{\pi }{\lambda {k}_{b}t}\right)}^{1/2}\frac{{H}_{\text{AB}}^{2}}{\hslash }\text{exp}\left(-\frac{\Delta {G}^{\ddagger }}{{k}_{b}T}\right)$$

where *k*_hop_ is the bimolecular charge transfer rates between radicals and oxidized cation, λ is the reorganization energy, *k*_b_ is the Boltzmann constant, *T* is the temperature, *H*_AB_ is the electronic coupling, ℏ is the reduced Planck constant, ΔG^⧧^ is the activation energy for the transition state [36–40]. The *k*_hop_ in above equation is emphasized by the proximity between the species in a solid-type electrolyte normally features its closer distance than that of the liquid-type electrolyte system. *K*. Sato et al. reported prediction of *H*_AB_ and Δ*G*^⧧^ value according to the distance of TEMPO, which demonstrate that the calculated and experimented value of H_AB_ increases and Δ*G*^⧧^ decreases in the reduced distance between radical sites of TEMPO molecules [36]. In our case, the dielectric screening effect of the solvent in the polymer electrolytes is reduced, leading to enhanced molecular interaction. Simultaneously, as the ratio of HT^•^ to HT^+^ approaches 1:1, it is anticipated that the distance between both HT^•^ and HT^+^ species with different charges can probabilistically become close. Eventually, there is an increase in the molecular interaction, thereby forming an effective conduction pathway and reducing the energy difference between reactants and the transition state. As a result, the accelerated charge hopping rate based on self-exchange reaction can lead to an increase in ionic conductivity (Fig. 3, purple arrow). Furthermore, Li^+^ ions, which are coupled species with HT, are transferred along the self-exchange reaction pathway within the both HT molecules, which can potentially induce the increase of the hopping rate of Li^+^ ions (Fig. 3, yellow arrow). Specifically, HT and (HT-Li)^+^ can form an intermediate state of bimolecular complex where a Li^+^ ion bridges the oxygen radicals of HTs. DFT calculations reveal that the binding energy of this Li^+^-bridged intermediate state is − 1.56 eV, which highlights the feasibility of this transition state in the self-exchange reaction (Fig. S12). Then, the HT species which was originally in the neutral form now generates a coordination complex with the lithium ion, resulting in a newly formed (HT-Li)^+^ species. Through consecutive formation and dissociation of coordination bonds, lithium ions can be transported, which potentially induce an increase in the hopping rate of Li^+^ ions by sequential self-exchange reactions.

These hypotheses highlight that **T-10** (N−O^•^: 36% and N^+^=O: 64%) and **T-20** (N−O^•^: 64% and N^+^=O: 36%) with an analogous ratio between N^+^=O and N−O^•^ in a series of HT_RPEs exhibit the higher ionic conductivity than other HT_RPE. However, **T-20** shows a decrease of the ionic conductivity due to the reducing the net carrier velocity of the system by the aggregation of free ions [41, 42]. To investigate the influence of the structural characteristics on ionic conductivity, **T-15** with nearly equal proportions of N−O^•^ and N^+^=O (49% and 51%, respectively) is analyzed by the high-resolution XPS spectra on N 1s (Fig. S13a, b). This ratio at **T-15** is similarly confirmed by the Raman spectra (Fig. S13c). Despite achieving the theoretically favorable ratio for charge carrier interactions, the ionic conductivity of **T-15** exhibits the decrease compared to **T-10** (Fig. S13d, e). This reduction in the ionic conductivity can be attributed to the crystallization and aggregation of HT, as evidenced by the XRD patterns (Fig. S13f). The crystalline structure formation potentially inhibits efficient self-exchange reactions between charge carriers, consequently leading to less effective ionic conduction channels through the matrix. This observation underscores that the optimal ionic conductivity is not solely determined by the ratio of charge carriers but is significantly influenced by the structural characteristics of the HT_RPEs. Eventually, **T-10** among our prepared series of HT_RPE displays the highest ionic conductivity by virtue of fast hopping process based on self-exchange reaction. Especially, solid-state **T-10** exhibits high ionic conductivity compared to that of previously reported Li ion-based solid-state polymer electrolyte (Table S1). Furthermore, the ionic conductivity of quasi-solid-state **T-10** surpasses those of previously reported redox polymer electrolytes with other additives such as halogen (KI, BMIMBr, LiBr and BMIMI), metal (Na_2_MoO_4_) and small organic molecules (HQ and BAAS) (Fig. 2g) [12, 15, 43–48]. Overall, these results highlight that **T-10** in a series of HT_RPEs can be a candidate for FSESDs with high electrochemical performance based on very fast self-exchange reaction.

### Mechanistic Investigation of Ion Conduction by Temperature-Dependent Studies

The dominant conduction mechanism of **T-10** could be identified by the shape of temperature-dependent ionic conductivity profile [49–51]. Typically, Volgel-Tammann-Fulcher (VTF) behavior, which is a nonlinear shape of plots between the logarithm of ionic conductivity and the reciprocal temperature, indicates ion conduction mechanism coupled with long-range motion by relaxation/breathing and/or segmental motion of a polymer chain. An Arrhenius behavior is characterized by a linear profile in a plot, which is observed in systems where ion hopping is distinct from long-range polymer motions or in highly crystalline rigid solid system. In the solid-state system, **T-0** and **T-1** show Arrhenius relationship by high crystallinity of polymer matrix at the temperatures below their *T*_*g*_ and a transition to VTF behavior at elevated temperatures above their *T*_g_ (Fig. S14a, b). **T-5** reveals ion transport behavior dominated by polymer chain motion, a characteristic typically observed in polymers maintaining a clear rubbery state (Fig. S14c). Since the temperature-dependent ionic conductivity profile of **T-10** and **T-20** closely fits a linear-least-square method, it appears to follow a quasi-Arrhenius behavior (Fig. S14d, e). Specifically, **T-10** and **T-20**, which maintain the closest 1:1 ratio between HT^+^ and HT^•^ at an appropriate concentration, can facilitate the transfer of the hopping process of charged species through a self-exchange reaction. Moreover, **T-10** and **T-20** developed an amorphous nature due to HT and LiClO_4_ acting as plasticizers clearly indicate that ion conduction through the segmental motion of the polymer cannot be ignored. Therefore, we suppose that the ion conduction mechanism of **T-10** is the hopping process of ionic species, based on a self-exchange reaction supported by the segmental motion of the polymer. In the **T-10**, the Li^+^ transport number was determined to be 0.47 through polarization measurements (Fig. S15). This is a reasonable value when compared to other polymer electrolyte systems incorporating various additives, particularly considering our unique charge transport mechanism where both HT/HT^+^ self-exchange reactions and Li^+^ migration contribute to the overall ionic conduction process (Table S1). Furthermore, temperature-dependent ionic conductivity analysis was conducted on **T-0** and **T-10** in their quasi-solid state to investigate the distinct effect of HT under fully plasticized conditions (Fig. S16). Although **T-0** demonstrates VTF behavior attributed to water-induced polymer chain plasticization, ion hopping through self-exchange reaction of HT remains the dominant transport mechanism in **T-10**. This result clearly demonstrates that the ion conduction in **T-10** predominantly relies on the radical hopping process through self-exchange reactions, rather than the polymer chain motion, even in the presence of strong plasticizers such as water. The persistence of this hopping mechanism, regardless of the physical state of polymer, highlights the robust and efficient nature of HT-mediated ion transport in our system. Furthermore, the activation energy calculated from the Arrhenius equation indicates the minimum energy required to transport the charge for the redox reaction, where the lower transition barrier usually allows for a high ionic conductivity. Notably, the activation energy for **T-10** was calculated to be 0.13 eV in the quasi-solid state and/or 0.20 eV in the solid-state. Especially, the activation energy of quasi-solid-state T-10 is a significantly lower value than that of the previously reported halogens or the small molecular organics (Fig. 2h and Table S2). These results suggest that **T-10** has a smaller transition barrier than the conventional redox additives, mainly owing to the self-exchange reaction based on excellent solubility of HT [12, 15, 52–55 ].

### Design of Fiber-Shaped Energy Storage Devices and Their Electrochemical Performance

**Fig. 4:**
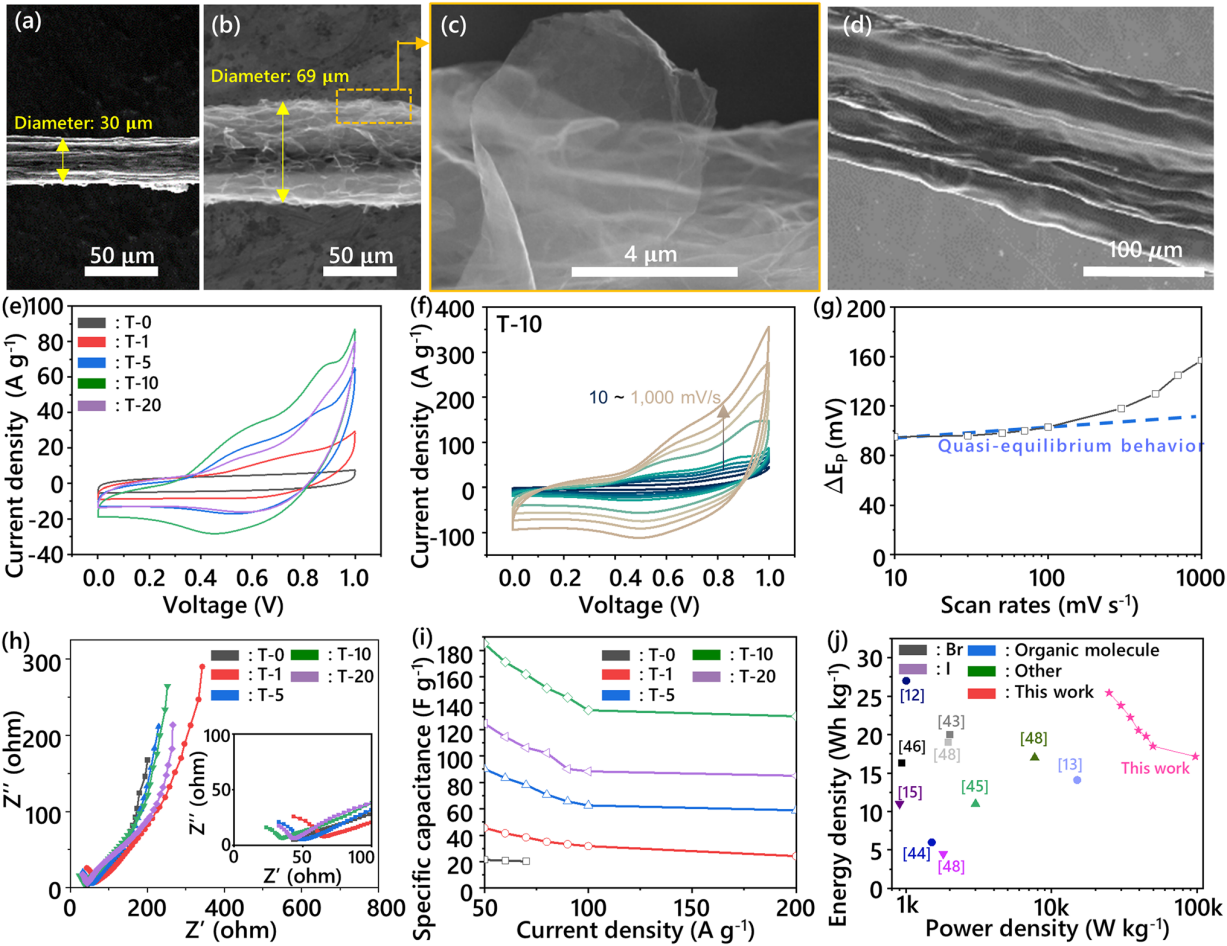
SEM top-view image of **a** CNTF, and **b** VG@CNTF. High-resolution SEM image of **c** VG@CNTF, and **d** FSESDs coated with T-10. **e** CV profiles of the RPE series at a scan rate of 100 mV s^−1^. **f** CV profiles of T-10 at the scan rates from 10 to 1,000 mV s^−1^. **g** Peak separation of CV profiles in T-10. **h** Nyquist plots (inset. high-frequency region). **i** Specific capacitance versus current densities of the RPE series. **j** Ragone plots with the redox polymer electrolytes series

As briefly mentioned, a carbon-based fiber electrode designed for FSESDs should provide plenty of sites for faradaic reactions, along with the presence of a redox additive to give a synergetic impact. A stepwise variation on the morphology of our FSESD following each fabrication step was observed by SEM. The morphology of the liquid crystal spun CNTF, with a diameter of 30 $\upmu$m, located at the core of the fiber electrodes exhibits a nonporous and highly packed structure with densified single-walled carbon nanotube (SWNT) bundles (Figs. 4a and S17a). The specific mechanical strength and the specific electrical conductivity of the core fiber showed an outstanding performance of 1.21 N tex^−1^ and 2945 S m^2^ kg^−1^, respectively (Fig. S17b). To enhance the compatibility of the fiber electrode with the HT_RPE for better accessibility of ions and HT, the VG@CNTF was prepared by the stereoscopically composited method via an electrophoretic deposition process. The resultant VG@CNTF with a diameter of 69 $\upmu$m, which shows a 3D-open porous structure with plenty of oxygen functionalities and exposed edge planes, is believed to be beneficial for the faradaic reactions (Fig. 4b, c). The SEM surface image of the FSESDs with **T-10** reveals that HT_RPE forms a uniform and continuous coating layer over the VG@CNTF electrode surface, highlighting complete coverage without any exposed areas (Fig. 4d). The nonporous and compact structure of the HT_RPE layer suggest intimate contact between the **T-10** and electrode, which is essential for efficient charge transfer at the interface.

The electrochemical characteristics of the FSESD with variable amounts of HT were then measured via CV at a scan rate of 100 mV s^−1^ (Figs. 4e and S18). The conventional polymer electrolyte system without a redox additive, namely **T-0**, exhibited capacitive behavior only from physical adsorption/desorption processes of ions at the interface of electrode/electrolytes. **T-1** with a small amount of HT shows higher electrochemical performance than that of **T-0**, due to its increased ionic conductivity, even though a distinct characteristic peak of HT is not clearly displayed. On the other hand, FSESDs, fabricated with **T-5**, generally displayed pseudocapacitive characteristics with a redox voltage range of 0.4–0.8 V, which corresponds to a reversible faradaic reaction associated with HT. The largest electrochemically active area was observed in **T-10**, which is the superlative electrochemical performance in FSESDs using polymer electrolytes. As discussed earlier, this is believed to be enabled by a synergetic effect of the excellent compatibility between the components, and a high level of ionic conductivity of our HT_RPE system. As mentioned before, an excessive HT concentration in the HT_RPE system (**T-20**) resulted in an inferior ionic conductivity as well as its electrochemical performance. To further investigate the origin of the high electrochemical performance of the **T-10**, CV analysis at ultra-fast scan rates and electrochemical kinetics are performed systematically. Notably, redox reaction of **T-10** is well maintained even at the scan rate of 1,000 mV s^−1^, which highlights the high level of ionic conductivity of **T-10** as well as the fast charge transfer at the interface between fiber electrode and **T-10** (Fig. 4f) Especially, the Δ*E*_*p*_, which represents the separation between cathodic and anodic peak potentials, was small (approximately 90 mV at scan rates ≤ 0.1 mV s^−1^). This small separation indicates that a highly reversible faradaic process occurs at the peak potential and that a clear quasi-equilibrium state exists (Fig. 4g). The electrochemical impedance spectroscopy (EIS) measurements were conducted to investigate the interaction between fiber electrode and polymer electrolyte in the FSESDs with different HT concentrations (Fig. 4h). The Nyquist plots show three distinct components of the bulk resistance (*R*_b_), charge transfer resistance (*R*_ct_), and diffusion resistance (*R*_D_). In the low-frequency region, **T-0** without HT delineates lower diffusion resistance than HT_RPEs due to the larger size of HT compared to the lithium ion. In the high-frequency region, the bulk resistance of **T-0** exhibits a relatively high value (*R*_b_ = 40 Ω) (Fig. 4h, inset)**.** Upon incorporation of HT, the bulk resistance of HT_RPEs decreases significantly, reaching its minimum value (*R*_b_ = 8.5 Ω) at the optimal HT concentration (**T-10**), which can be attributed to enhanced ionic conductivity. The charge transfer resistance of **T-0** is not observed in the high-frequency region; however, HT_RPEs show definite semicircles related to the charge transfer of HT. The charge transfer resistance reveals a declining tendency as the concentration of HT increases from **T-1** (*R*_ct_ of **T-1**: 45 Ω, *R*_ct_ of **T-5**: 32 Ω, *R*_ct_ of **T-10**: 25 Ω, and *R*_ct_ of **T-20**: 24 Ω). The progressive elevation in the concentration of HT results in a high amount of HT molecules in the electric double layer region at the interface of the fiber electrode. This indicates a higher probability of HT encounters at the electrode surface, suggesting that charge transfer reactions can occur more readily. Therefore, the overall charge transfer process is facilitated, leading to a reduction in charge transfer resistance. The specific capacitance of the FSESDs with different concentrations of HT was calculated from the discharge branch of GCD profiles (Fig. S19). The specific capacitance reveals that **T-10** (183 F g^−1^ at 50 A g^−1^) exhibits an approximately 8 times higher electrochemical activity than that of the conventional polymer electrolyte system (**T-0**, 21 F g^−1^ at 50 A g^−1^) (Fig. 4i). The FSESDs made up of excess HT (i.e., **T-20**) showed a reduced specific capacitance (124 F g^−1^ at 50 A g^−1^). Furthermore, **T-10** exhibited the highest rate capability of 71% at 200 A g^−1^ compared with the other FSESDs, mainly owing to fast ionic transport through the optimized performance. The electrochemical performance of our FSESDs was compared with previously reported systems utilizing redox-active electrolytes in a Ragone plot (Fig. 4j). The FSESD based on T-10 showed superior performance over conventional redox electrolyte systems incorporating halogens (Br, I), metal ions (Na_2_MoO_4_), or organic molecules (hydroquinone), achieving an energy density of 25.4 Wh kg^−1^ at a power density of 25,000 W kg^−1^. This enhancement can be attributed to the unique self-exchange reaction mechanism of HT and its optimal ratio with LiClO_4_. Most notably, our device maintained an impressive energy density of 17.1 Wh kg^−1^ even at an ultrahigh power density of 97,000 W kg^−1^, demonstrating an exceptional rate capability compared to other redox-active systems. Furthermore, when compared to previously reported energy storage devices using similar electrode configurations (Table S3), our HT_RPE system shows distinct advantages. While conventional polymer electrolyte systems (H_2_SO_4_/PVA, H_3_PO_4_/PVA, or KOH/PVA) with various carbon-based fiber electrodes typically deliver energy densities between 4 and 12 Wh kg^−1^, our system achieves more than twice these values. Although a MoS_2_/r-GO/CNT fiber with a H_2_SO_4_/PVA electrolyte showed a comparable energy density (26.4 Wh kg^−1^), it could only operate at a much lower power density (4,000 W kg^−1^). This exceptional overall performance stems from the synergistic combination of the enhanced ionic conductivity (73.5 mS cm^−1^) achieved through optimized HT concentration and the excellent interfacial compatibility between the VG@CNTF electrode and HT_RPE [55–58].

### Thermal Stability and Mechanical Durability of FSESDs

**Fig. 5:**
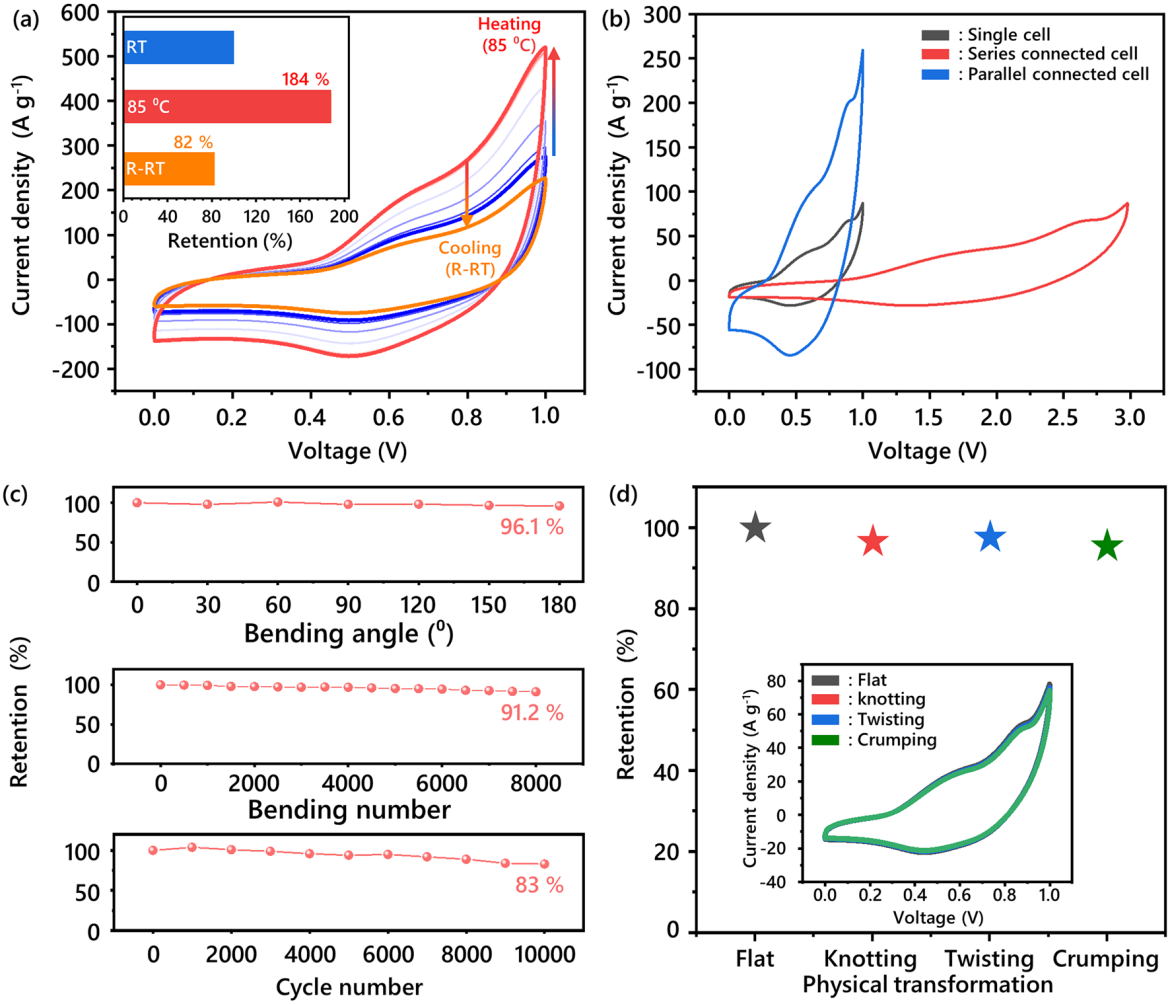
**a** Temperature-dependent CV profiles of T-10 from room temperature to 85 °C, and cooled back to the room temperature (inset. capacitance retention at each stage). **b** CV curves of a single device, series, and parallel connected devices. **c** Stability in terms of bending angle, bending cycles and charging/discharging cycles. **d** Flexibility testing of FSESDs to various physical deformation (inset. CV profiles correspond to various physical deformations)

In the discussion made above on the UV–Vis results, radical sites in the HT_RPE were found to be maintained even at a temperature of 85 °C. Therefore, the thermal stability of the FSESD using **T-10** was further evaluated by conducting CV measurements at elevated temperatures (Fig. 5a). The measurement was carried out by increasing the temperature at intervals of 10 °C from room temperature, where 20 min of equilibration time was applied for each step. The CV area of the FSESD was expanded by 184%, which is likely to be from the enhanced ionic conductivity of the HT_RPE. It is considered that the faster mobility of charges at high temperatures was primarily responsible for this enhancement, as well as the activated motion of the charges and/or the faster segmental motions of the polymer chains [55]. When cooled back to the room temperature, the device showed a high capacitance retention of 82% and unchanged peak position from HT, highlighting the thermal stability of HT (Fig. 5a, inset).

To demonstrate the practicality of our FSESD system, we tested both the flexibility of the device at various type of deformations. The FSESD displayed stable connection in either a parallel or a series configuration during and after the test and showed a good retention overall. It signifies the durability and adaptation of our fabricated device, assembled on demand for a variety of device form factors (Fig. 5b). In detail, the FSESD maintained nearly the same electrochemical performance under the bending angle from 0° to 180° (Fig. 5c, top panel) and exhibited high electrochemical retention of 91.2% after repeated bending cycles of > 8,000 (Fig. 5c, middle panel). Further, we tested the electrochemical retention over various harsh physical deformations, such as knotting, twisting, and crumpling (Fig. 5d). Among these, it maintained nearly identical electrochemical performances over 10 cycles of twisting, and the crumping of the FSESD exhibited an extraordinary capacitance retention of 95.3%. The FSESD undergoing repetitive charge/discharge cycles over 10,000 times at a current density of 50 A g^−1^ showed high capacitance retention of 83% (Fig. 5c, bottom panel). Especially, both N^+^=O and N−O^•^ species of **T-10** after the cycling test represent minimal transition (N−O^•^: 64% to 69%, N^+^=O: 36% to 31%), which highlight well-preserved chemical state of HT_RPEs during the charge/discharge process (Fig. S20). These impressive cycle life results demonstrate that the chemical stability of the redox species within HT_RPEs, combined with the robust durability of the carbon-based fiber electrode, contributes substantially to the overall excellent electrochemical performance of our system [59–61]. Comprehensively, FSESD using **T-10** displays an optimal electrochemical performance owing to the high accessibility of both the ions and HT to the core, and the high interfacial compatibility between the VG@CNTF and the HT_RPE. Furthermore, the electrochemical characteristics and performance of our FSESDs in general demonstrate the possibility toward various types of flexible electronics assembled on demand.

## Conclusion

We have successfully designed an inventive redox polymer electrolyte with a high ionic conductivity by a distinctive ion conduction mechanism. HT in the HT_RPE enhances polymer chain mobility by expanding amorphous domains and facilitating glass-to-rubber transition, thereby enabling ion transport via segmental motion at ambient temperature. Furthermore, the self-exchange reaction of HT in HT_RPE system has been proposed as a charged species transfer pathway for the high ionic conductivity. As a result, the **T-10** achieved an outstanding ionic conductivity (73.5 mS cm^−1^) based on a lower activation energy (0.13 eV) via the facile hopping of the charges supported by virtue of the segmental motion of the polymer. HT serves as the replacement for pseudocapacitive materials in the composite fiber electrode. Thus, the FSESDs, fabricated with the **T-10** and an active material-free fiber electrode, exhibited an outstanding electrochemical performance of 25.4 Wh kg^−1^ at 25,000 W kg^−1^, together with a high cycle retention of 83% after 10,000 charge/discharge cycles. In the thermally and mechanically harsh conditions, FSESDs showed high capacitance retention (82% at recovery after exposure to 85 °C, and 91.2% after 8,000 bending cycles), which reveals the high mechanical and thermal durability of **T-10**. Building upon these promising results, expanding our studies to different TEMPO derivatives (e.g., 4-amino-TEMPO, 4-carboxy-TEMPO) and polymer matrices beyond PVA will provide deeper insights on the advancement of flexible energy storage devices and solid electrolyte systems.

## Supplementary Information

Below is the link to the electronic supplementary material.

Supplementary file1 (DOCX 9032 KB)
