# Supplementary material for: Organic Radical-Boosted Ionic Conductivity in Redox Polymer Electrolyte for Advanced Fiber-Shaped Energy Storage Devices
Source: Nanomicro Lett. 2025 Mar 13;17:185. doi: 10.1007/s40820-025-01700-9 (PMC11906932; doi:10.1007/s40820-025-01700-9)
Supplement: Supplementary file 1 — Supplementary file1 (DOCX 9032 KB) [file 40820_2025_1700_MOESM1_ESM.docx]

Supplementary Information for

**Organic Radical-Boosted Ionic Conductivity in Redox Polymer Electrolyte for Advanced Fiber-Shaped Energy Storage Devices**

Jeong-Gil Kim^a,b,+^, Jaehyoung Ko^a,+^, Hyung-Kyu Lim^c^, Yerin Jo^d^, Hayoung Yu^a^, Min Woo Kim^a,e^, Min Ji Kim^a,e^, Hyeon Su Jeong^a^, Jinwoo Lee^b,*^ Yongho Joo ^a,*^ and Nam Dong Kim^a,*^

^a^ Institute of Advanced Composite Materials, Korea Institute of Science and Technology, 92 Chudong-ro, Bongdong-eup, Wanju-gun, Jeollabuk-do 55324, Republic of Korea

^b^ Department of Chemical and Biomolecular Engineering, Korea Advanced Institute of Science and Technology, 291 Daehak-ro, Yuseong-gu, Daejeon 34141, Republic of Korea

^c^ Division of Chemical and Bioengineering, Kangwon National University, Chuncheon 24341, Republic of Korea

^d^ Kims Reference Corporation, 218 Gajeong-ro, Yuseong-gu, Dajeong 34129, Republic of Korea

^e^ Department of Materials Science & Engineering, Gwangju Institute of Science & Technology, Gwangju 61005, Republic of Korea

^+^ These authors contributed equally to this work.

*Correspondence authors. E-mail: [ndkim@kist.re.kr](mailto:ndkim@kist.re.kr) (Nam Dong Kim); [jwlee1@kaist.ac.kr](mailto:jwlee1@kaist.ac.kr) (Jinwoo Lee); [yjoo0727@kist.re.kr](mailto:yjoo0727@kist.re.kr) (Yongho Joo).

**Supplementary Figures and Table**


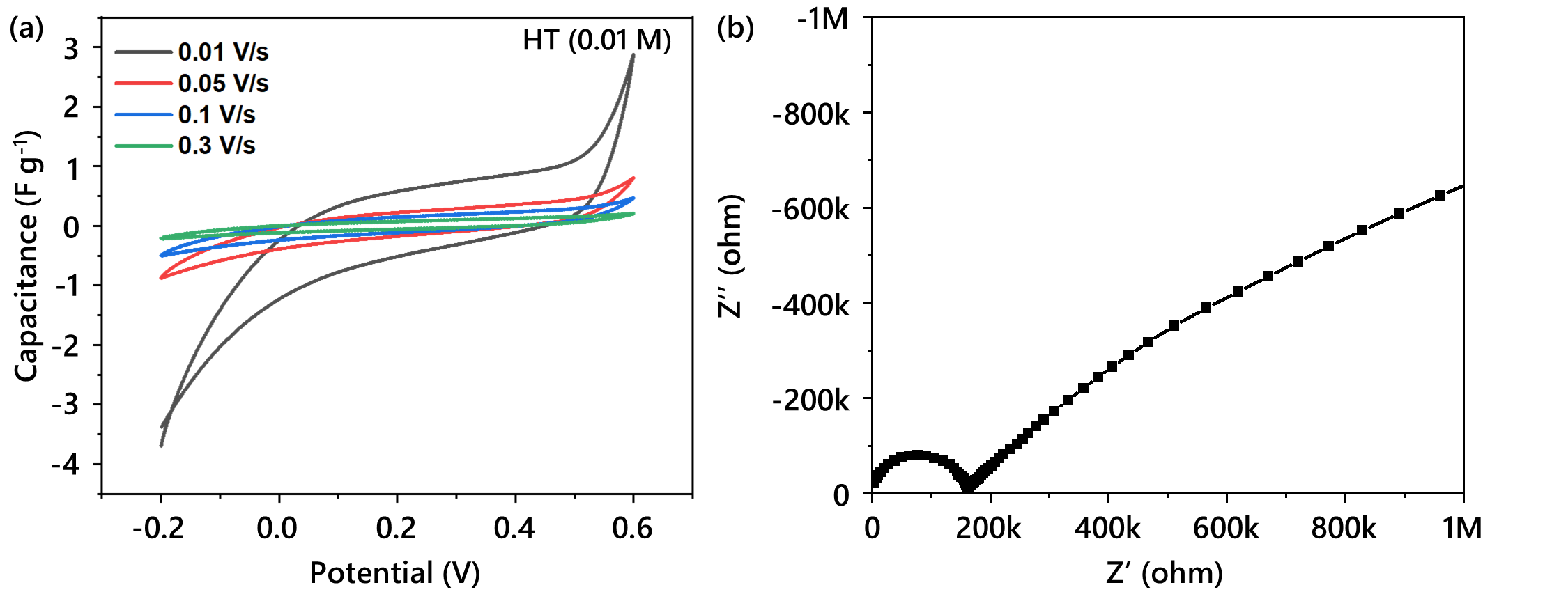


**Fig. S1** Electrochemical response of the aqueous redox electrolyte with 0.01 M HT (without salt). **a** CV profiles, and **b** a Nyquist plot from electrochemical impedance spectroscopy.


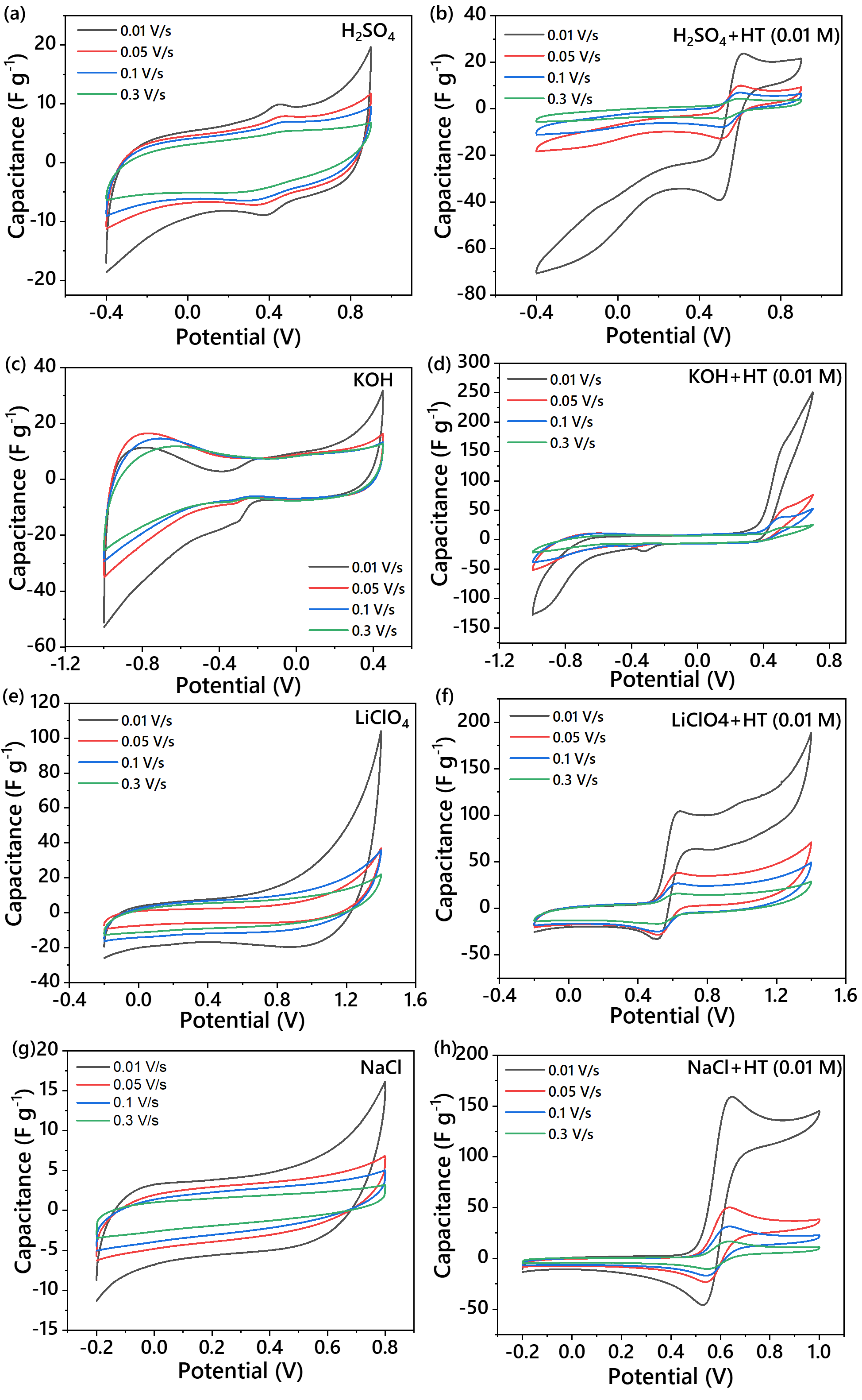


**Fig. S2** CV profiles of aqueous redox electrolyte with 0.01 M HT and 1 M salts. **a** H_2_SO_4_, **b** H_2_SO_4_ and HT, **c** KOH, **d** KOH and HT, **e** LiClO_4_, **f** LiClO_4_ and HT, **g** NaCl, and **h** NaCl and HT.


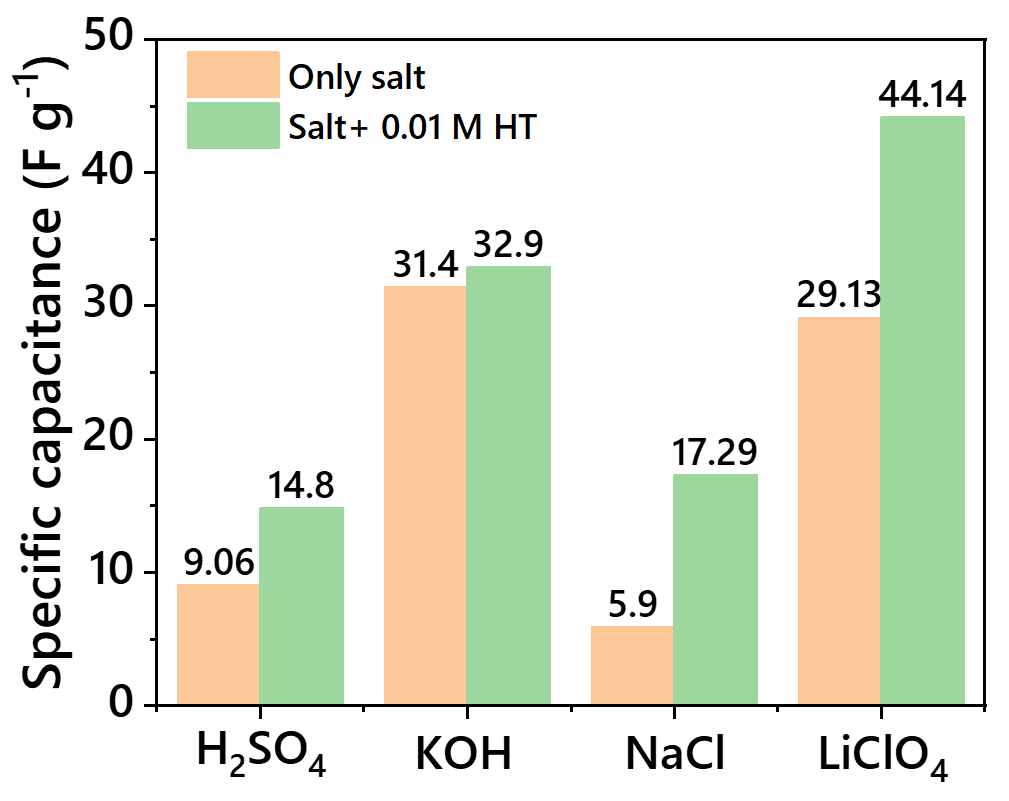


**Fig. S3** Specific capacitance of aqueous redox electrolyte with 0.01 M of HT and 1 M of salts.


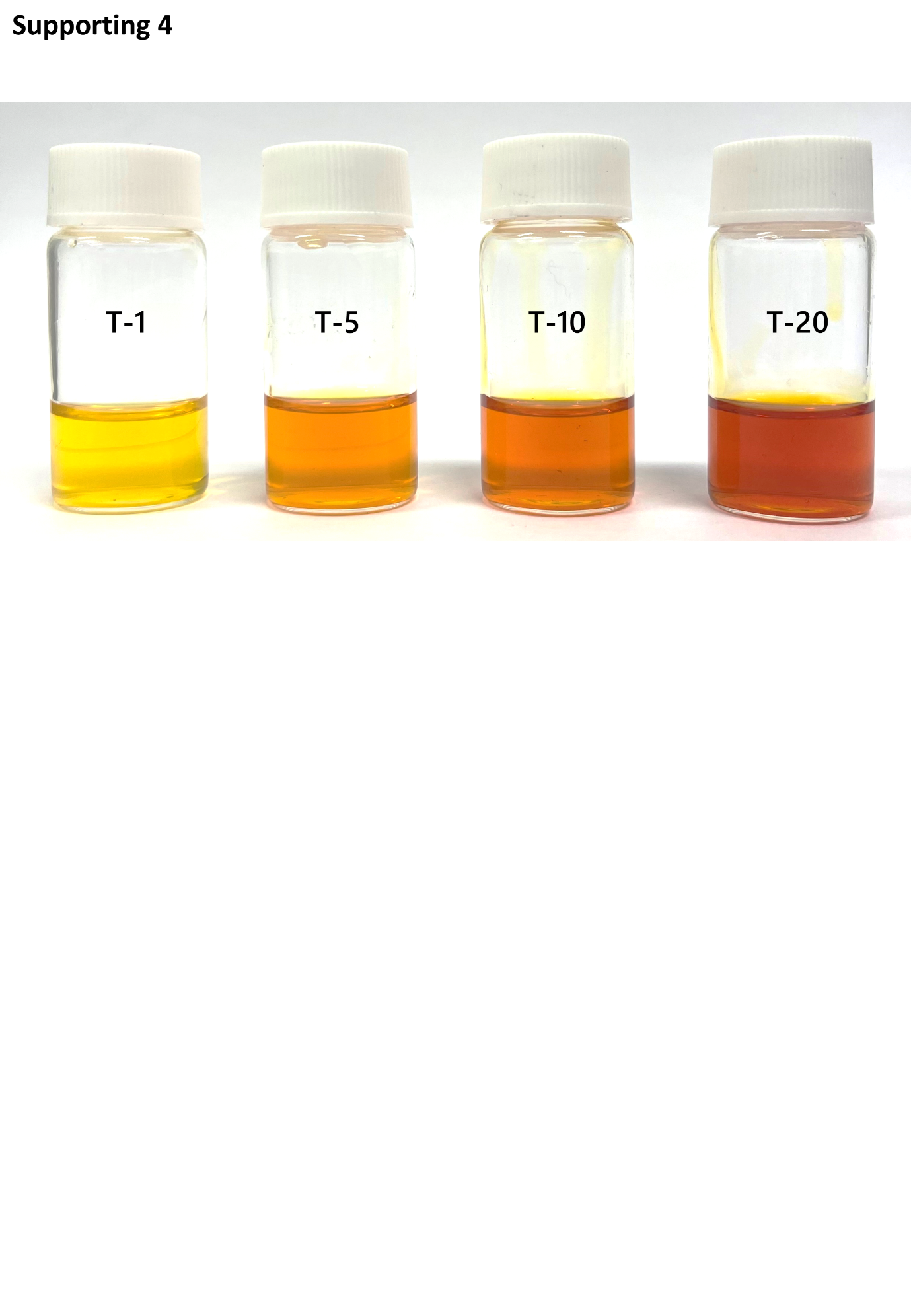


**Fig. S4** Photos of the RPEs utilized in this study.


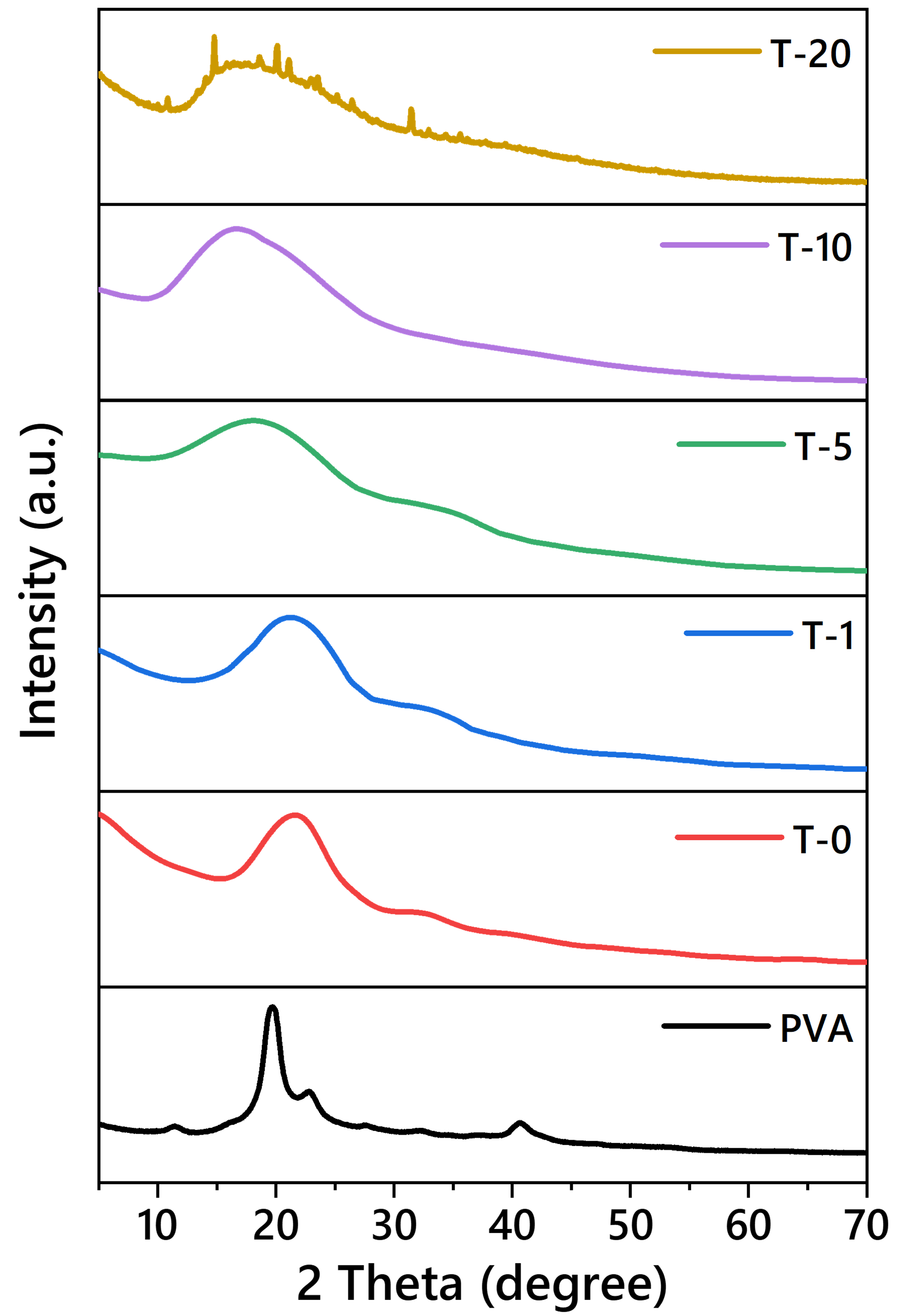


**Fig. S5** XRD pattern of pristine PVA, T-0, and HT_RPE systems.


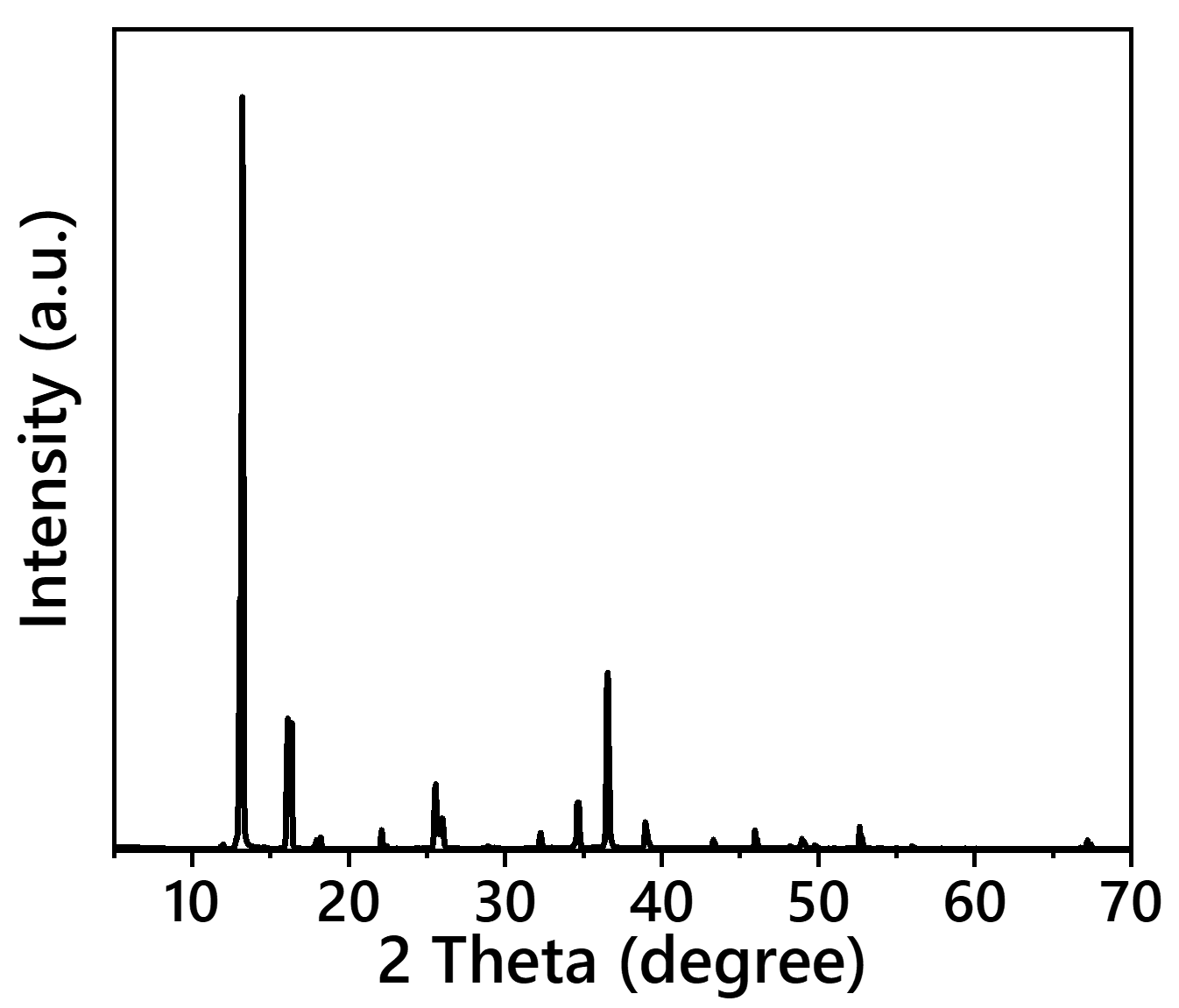


**Fig. S6** XRD pattern of pure HT.


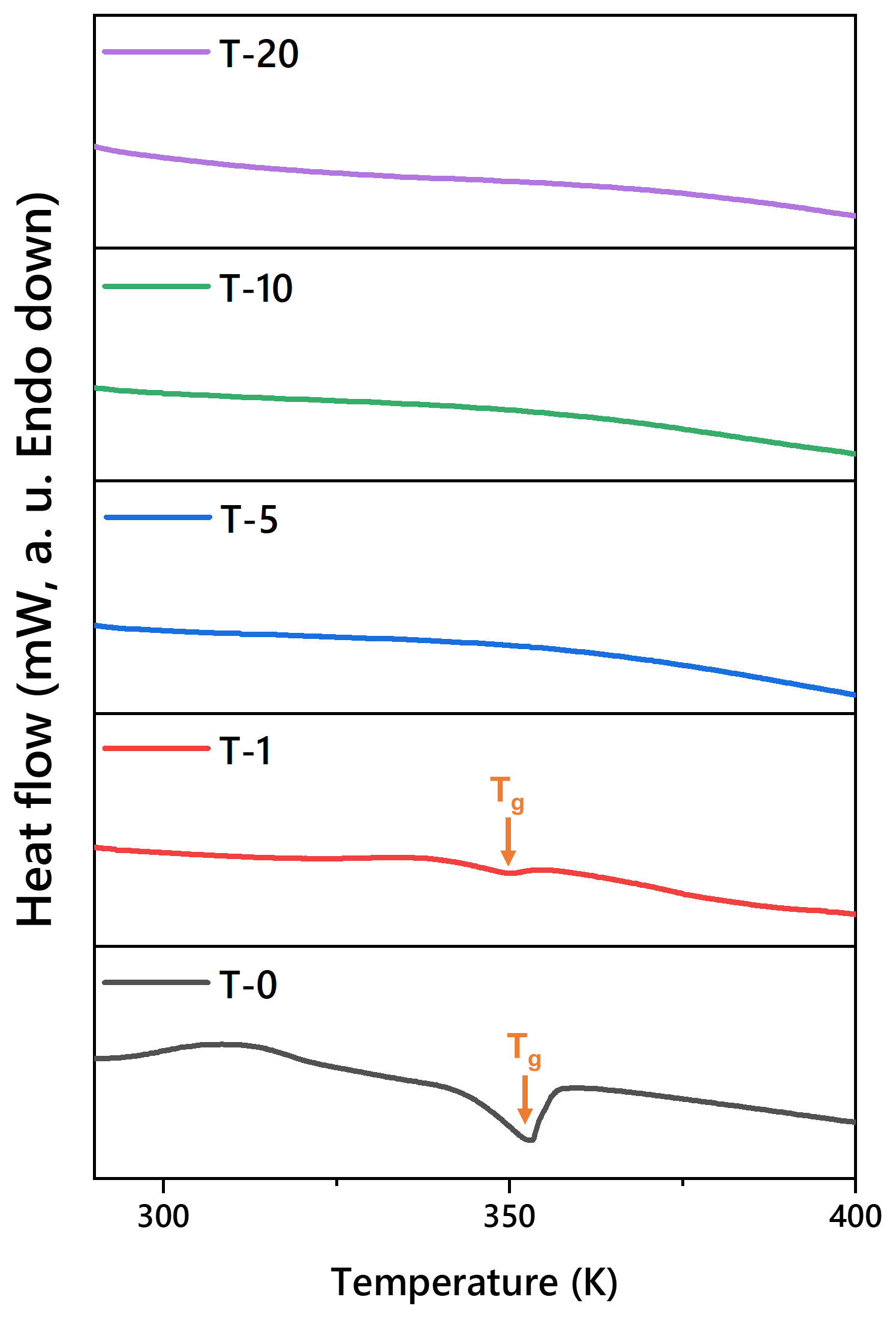


**Fig. S7** Differential scanning calorimetry profiles of samples for measurement of glass transition temperature


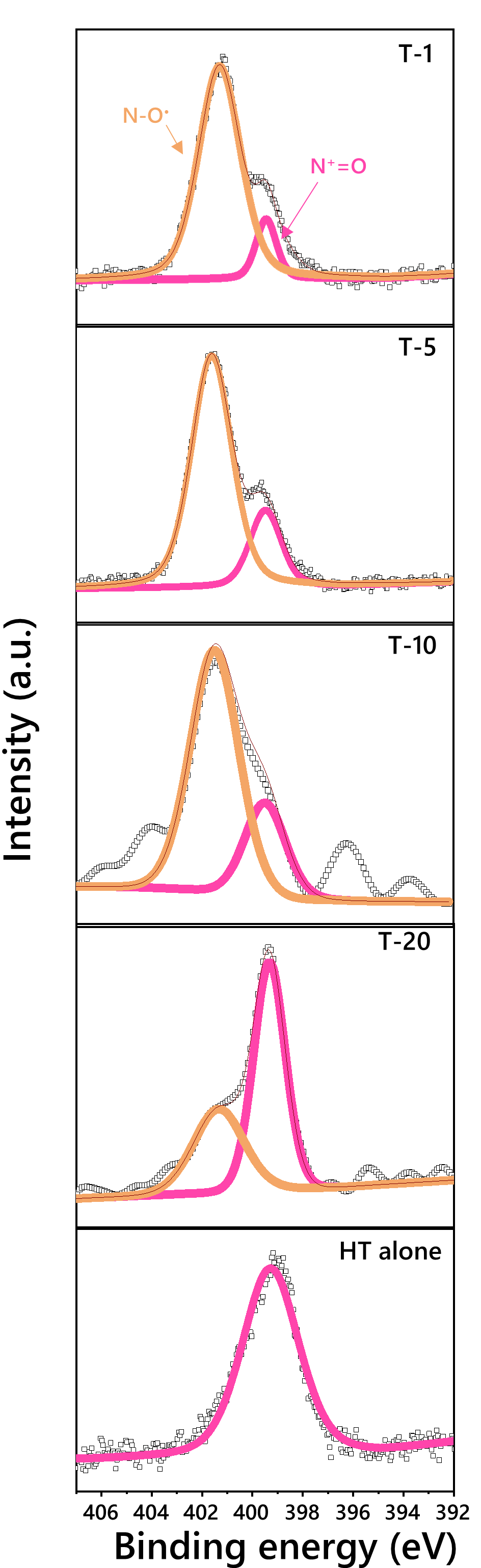


**Fig. S8** High resolution XPS spectra of pure HT and the RPE series from **T-1** to **T-20**.


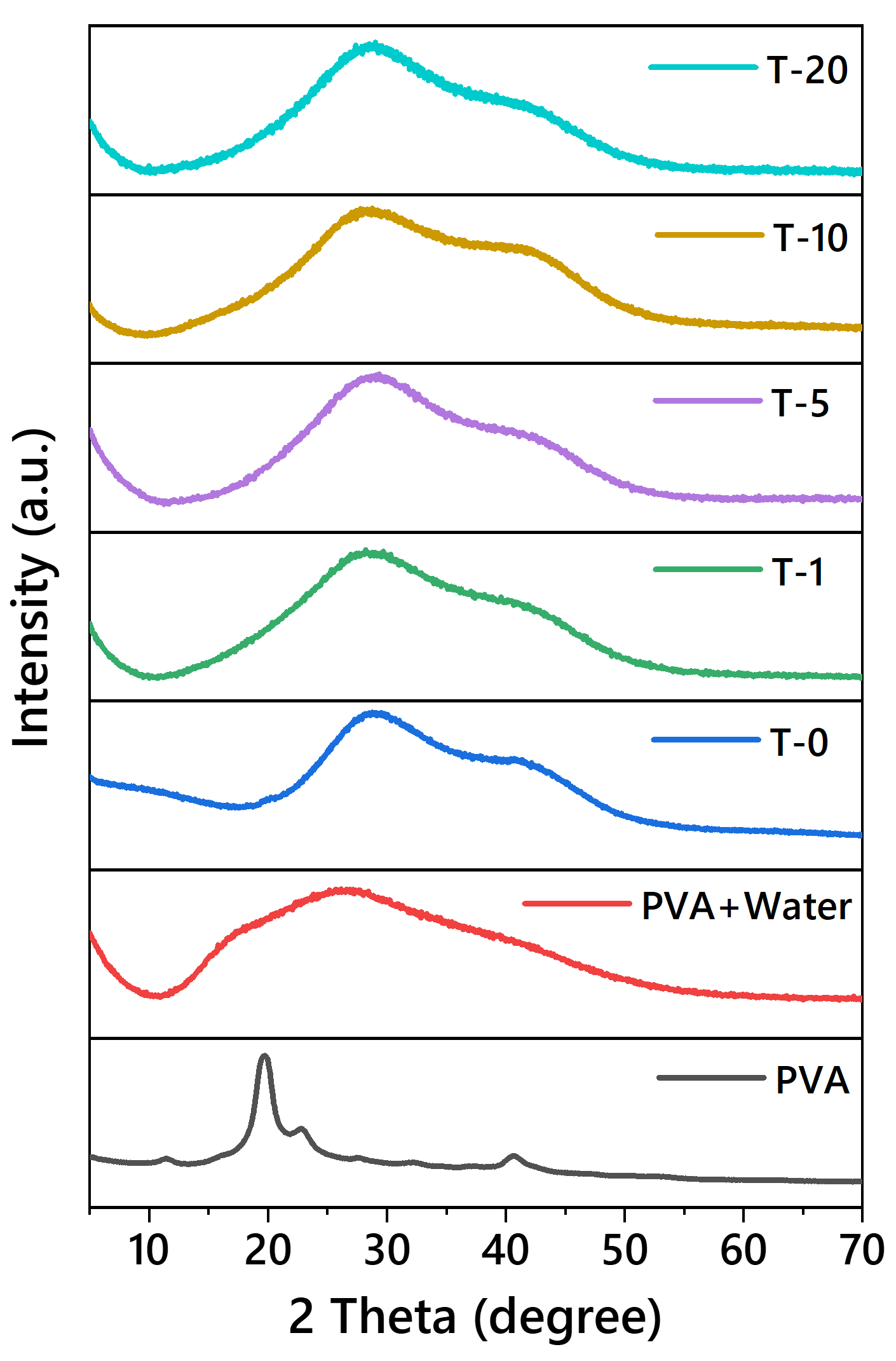


**Fig. S9** XRD spectra of pristine PVA, PVA added water and varying concentration of HT_RPEs in quasi solid state.


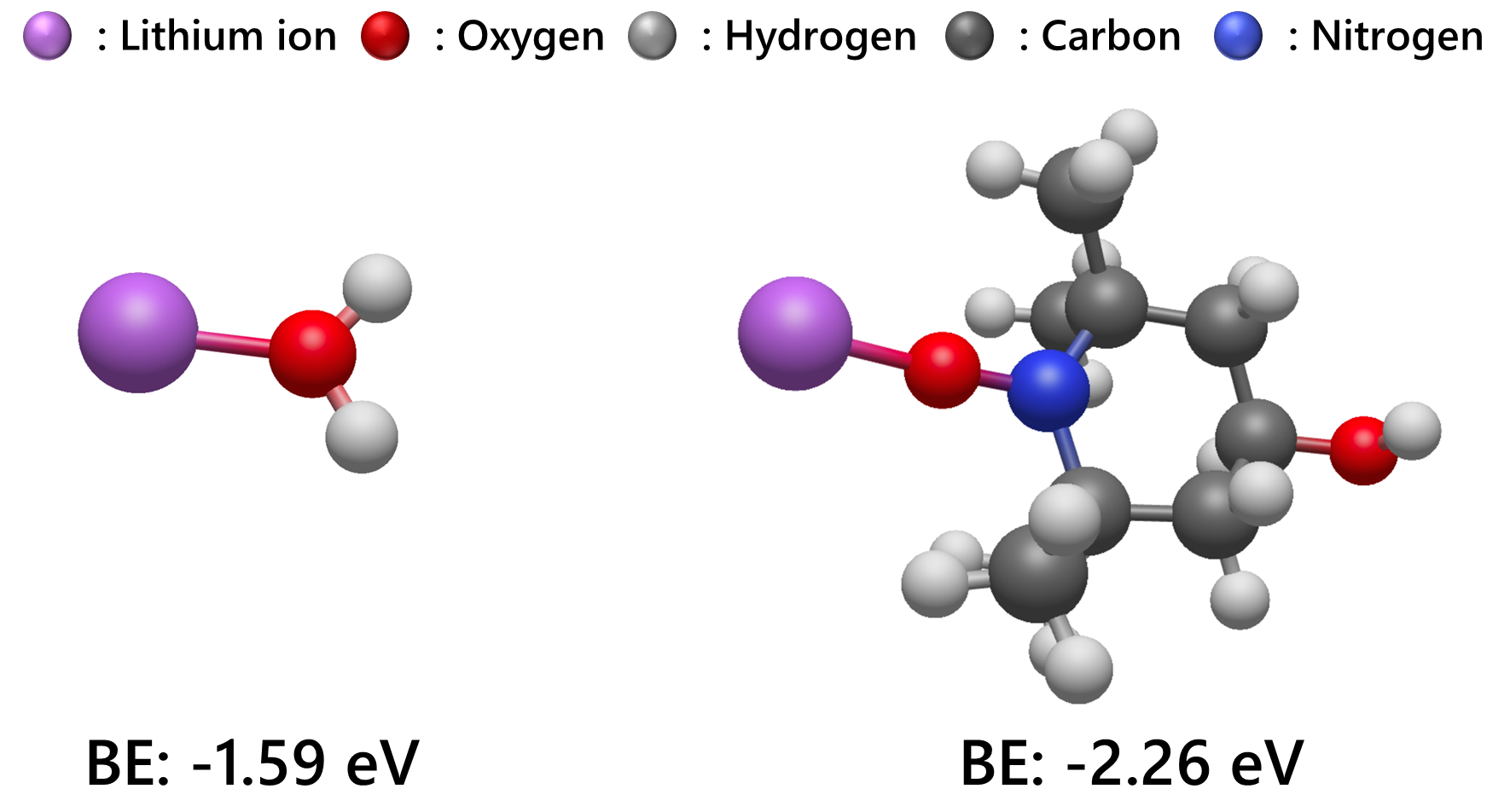


**Fig. S10** DFT-calculated binding energies (BE) between Li^+^ and different molecular species.


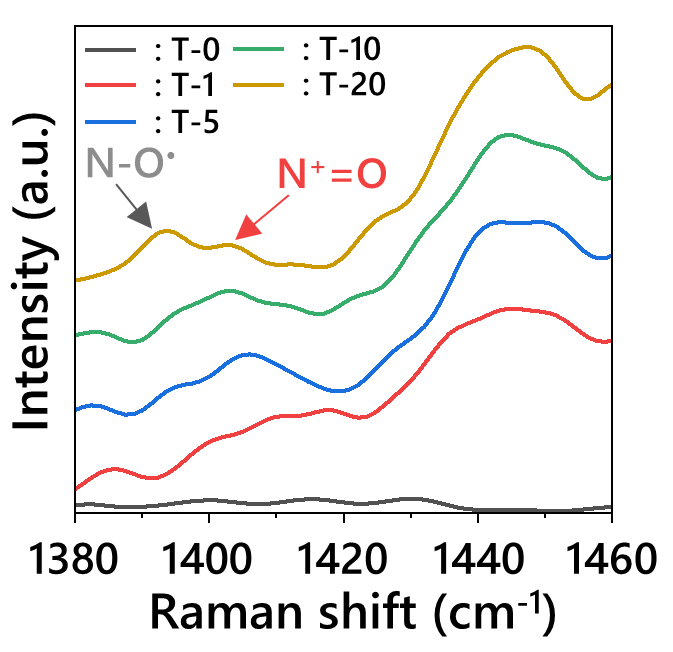


**Fig. S11** Raman spectra of samples with different HT concentrations.


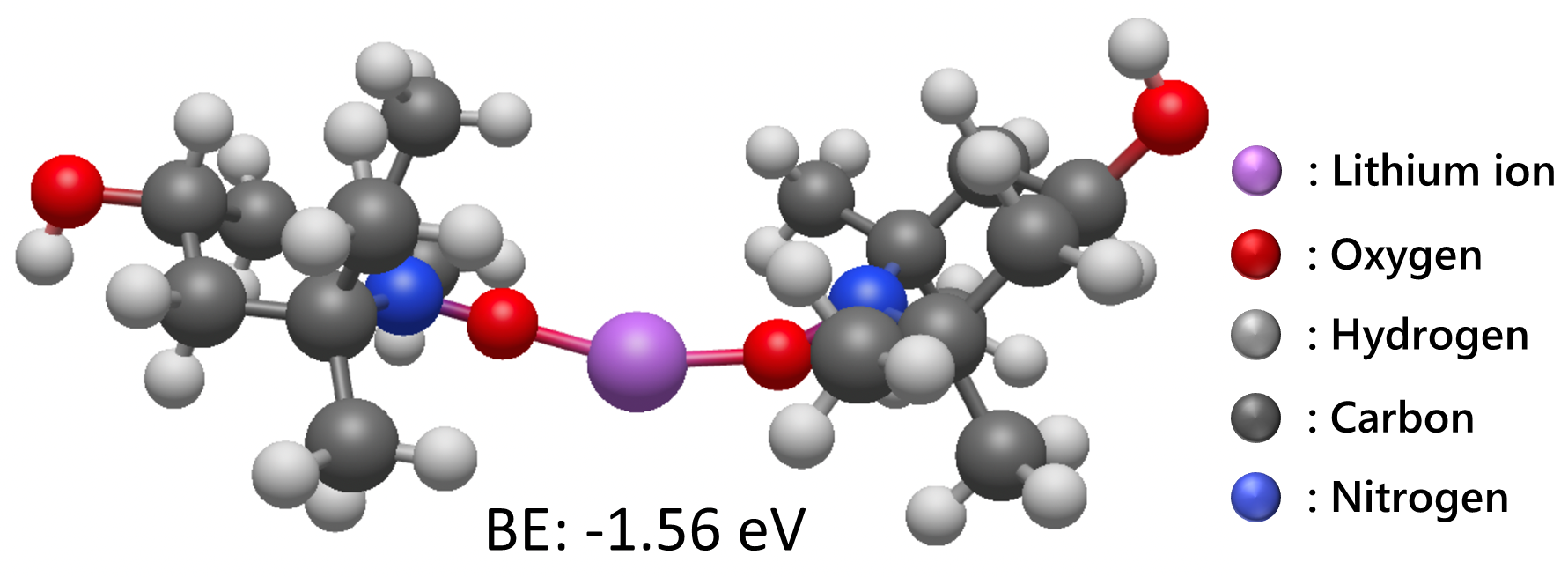


**Fig. S12** Binding energy (BE) of Li^+^ bridged between two HT molecules calculated by DFT


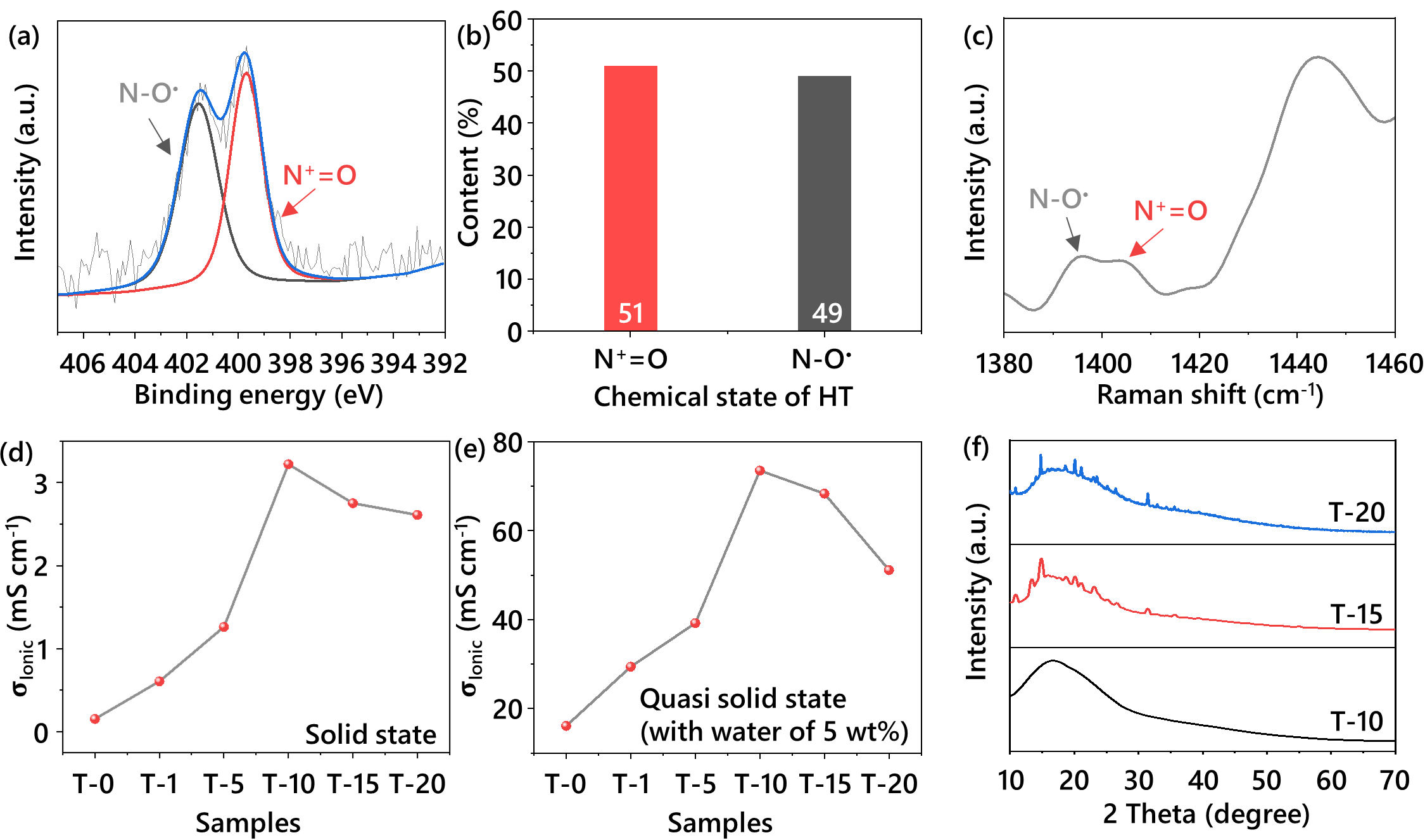


**Fig. S13 a** N 1s XPS spectra of T-15 showing the deconvolution of chemical states (N-O• and N^+^=O) in HT. **b** Relative content of N-O^•^ and N^+^=O chemical states obtained from XPS spectra. **c** Raman spectra of T-15 showing characteristic peaks corresponding to N-O^•^ and N^+^=O vibrations. **d** XRD patterns of T-10, T-15, and T-20 samples. **e** Ionic conductivity measurements in solid state and **f** quasi-solid state (with 5 wt% water content) for samples T-0 through T-20.


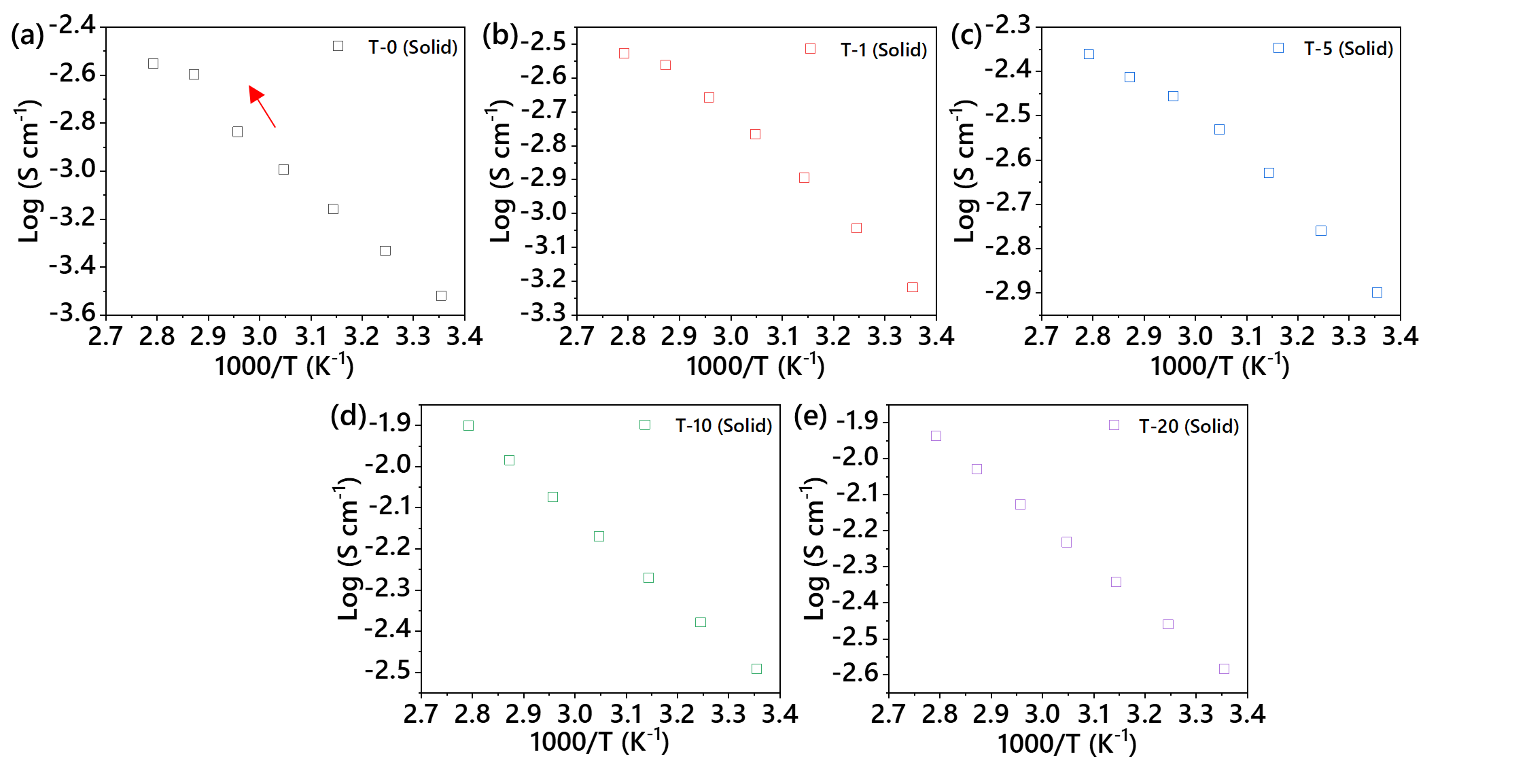


**Fig. S14** Temperature-dependent ionic conductivity of **a** T-0, **b** T-1, **c** T-5, **d** T-10, and **e** T-20


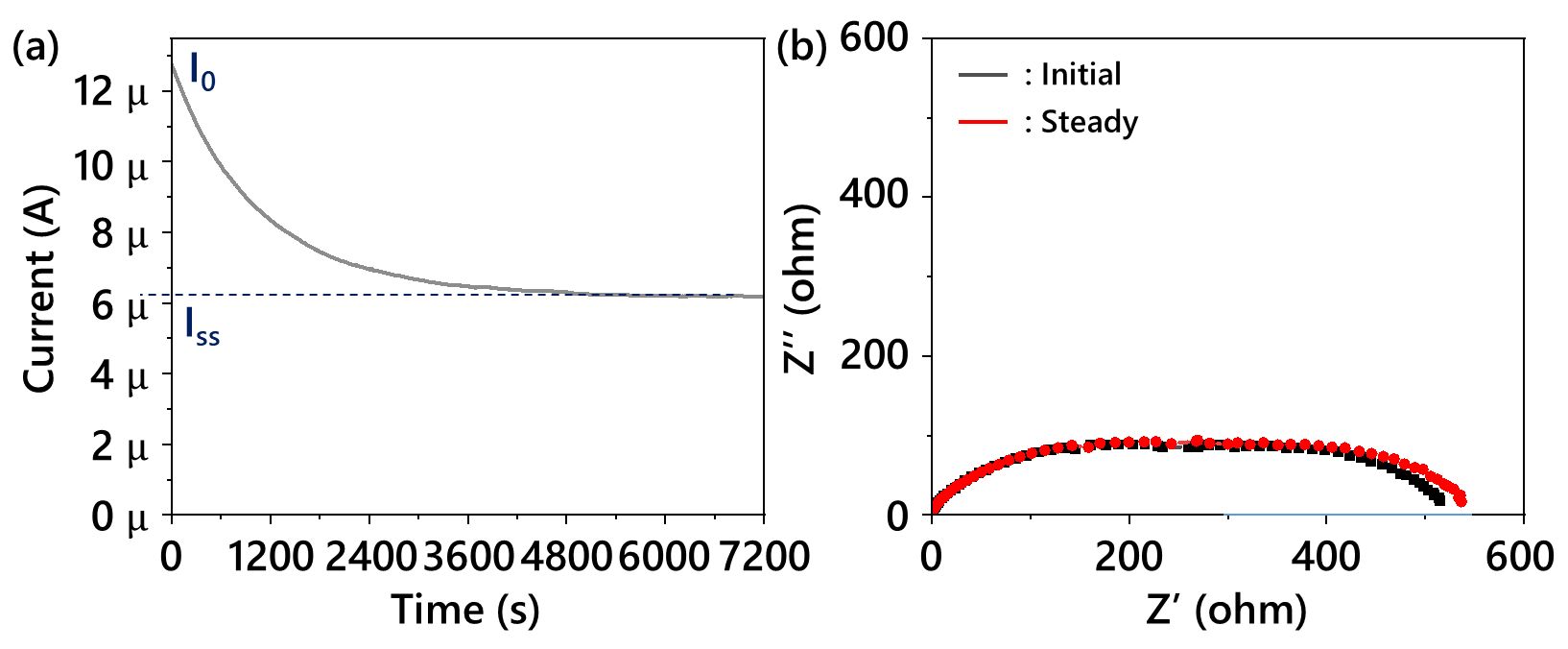


**Fig. S15** **a** Time-dependent current profile obtained from DC polarization measurements for determining Li^+^ transference number. **b** Nyquist plot exhibiting the impedance response before and after polarization.


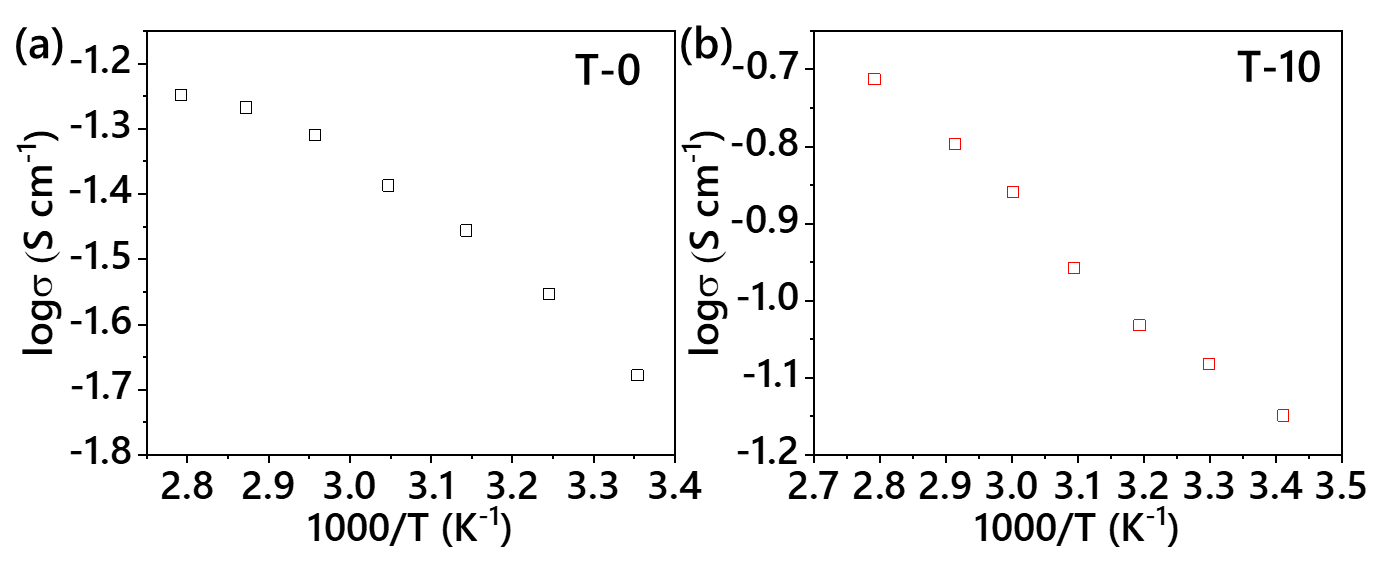


**Fig. S16** Ionic conductivity with respect to the temperature, derived from Rs in the Nyquist plots of **a** T-0 and **b** T-10


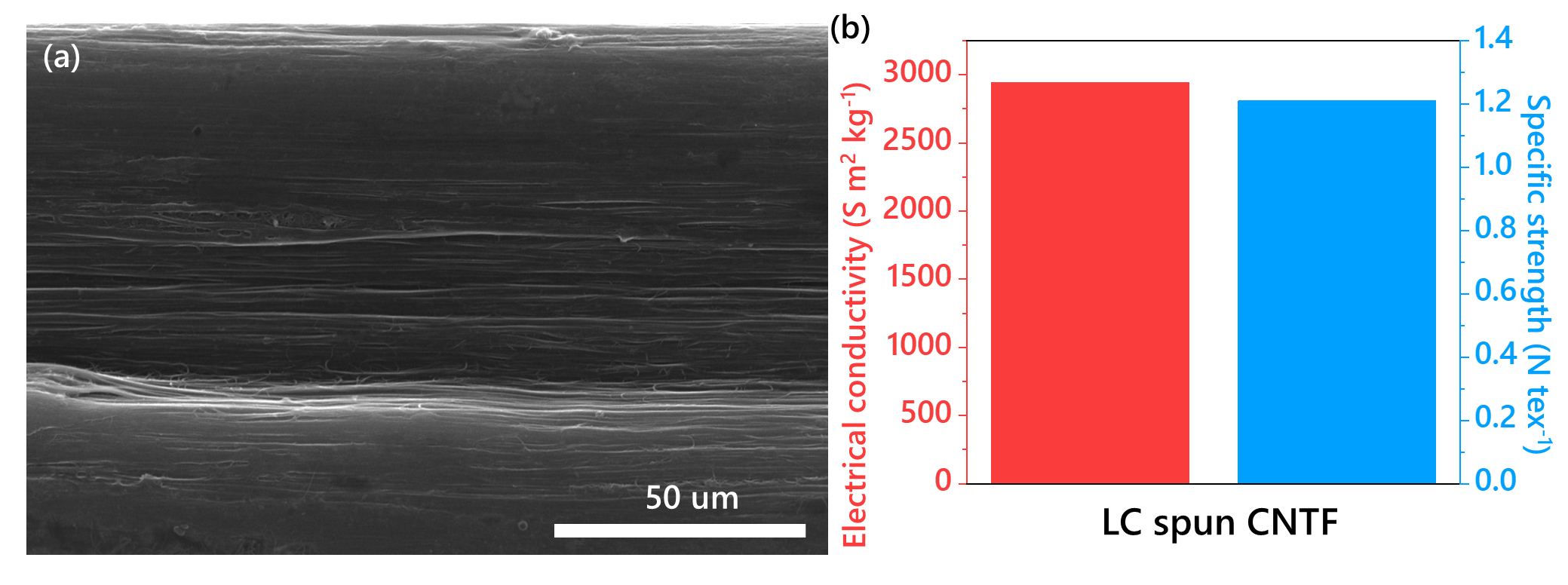


**Fig. S17** **a** High-magnification SEM image of the liquid crystal spun CNTF. **b** Electrical conductivity and specific strength of the liquid crystal spun CNTF.


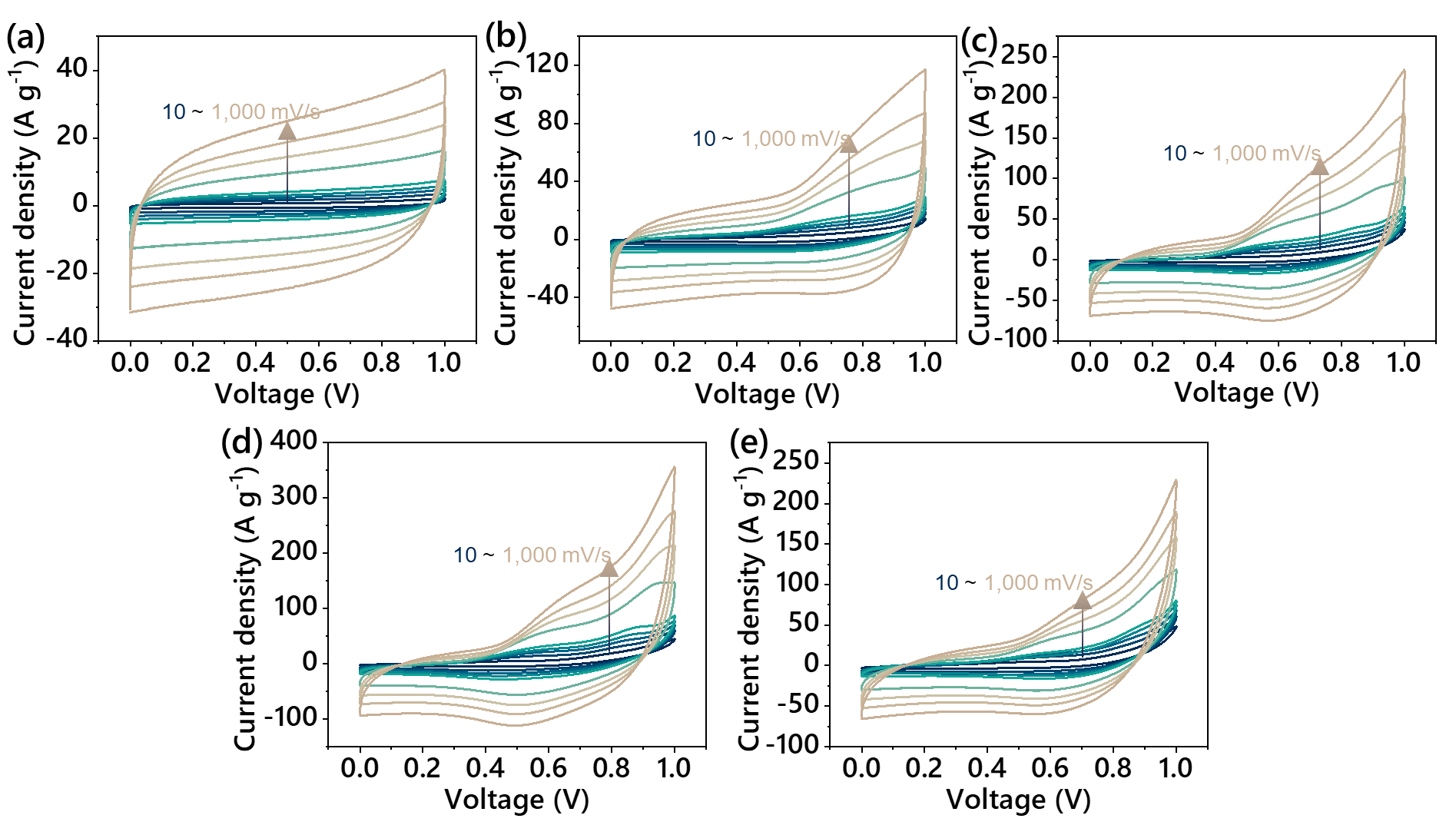


**Fig. S18** CV profiles at scan rates from 10 mV s^-1^ to 1,000 mV s^-1^ of **a** T-0, **b** T-1, **c** T-5, **d** T-10, and **e** T-20.


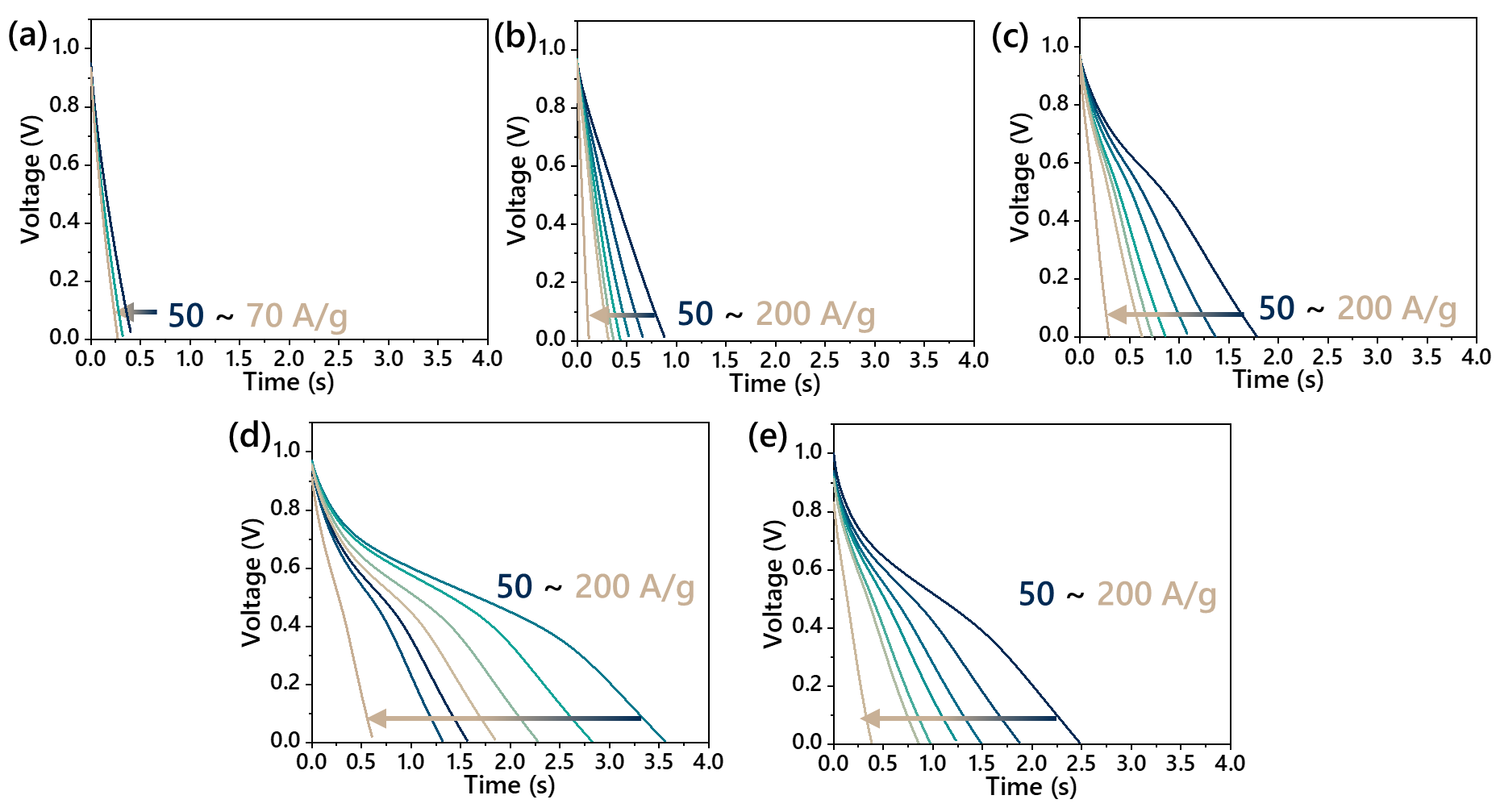


**Fig. S19** GCD profiles at current densities from 50 A g^-1^ to 200 A g^-1^ of **a** T-0, **b** T-1, **c** T-5, **d** T-10, and **e** T-20.


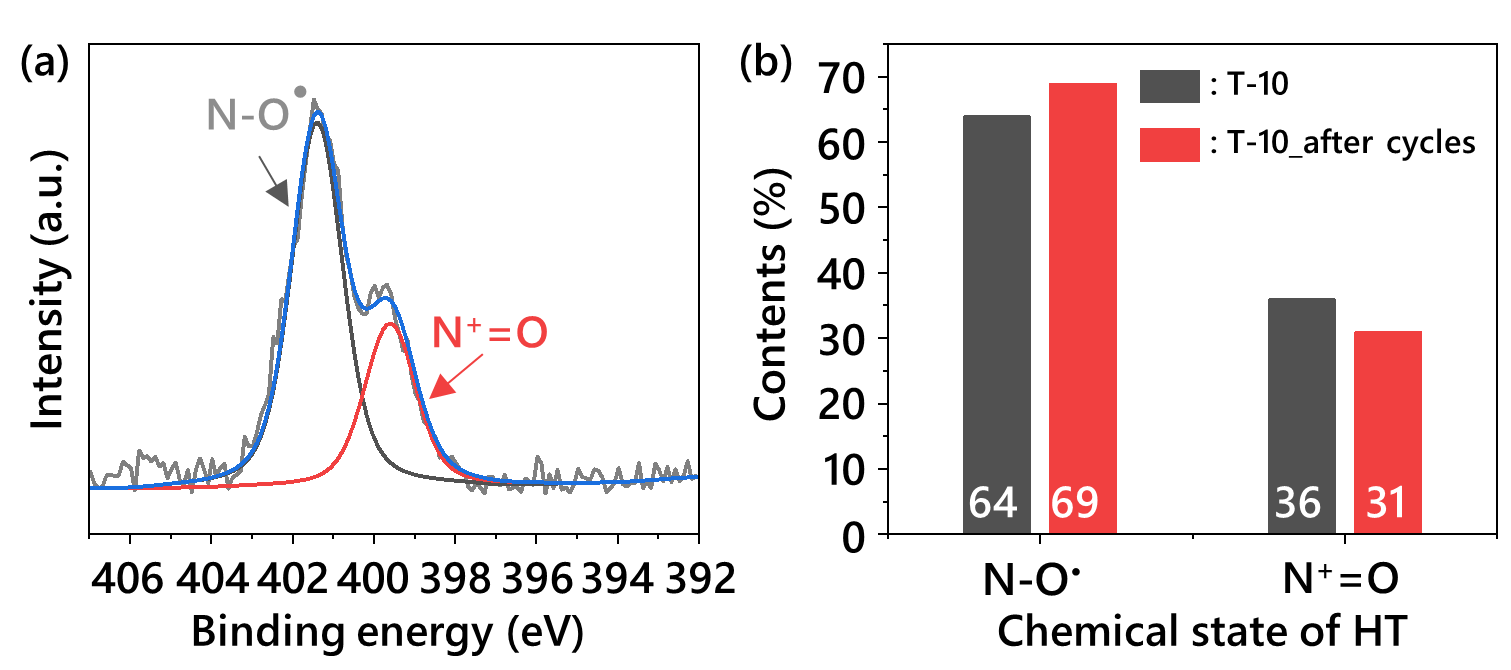


**Fig. S20** Chemical state analysis of HT in the T-10 before and after cycling test. **a** High resolution XPS N 1s spectra. **b** Relative contents of N−O^•^ and N^+^=O species quantified from peak deconvolution.

**Table S1** Ionic conductivity of varying previous studies

| Ref. | Polymers | Salts | Additives | Ionic conductivity  (mS cm^-1^) | t_Li_^+^ |
| --- | --- | --- | --- | --- | --- |
| [S1] | PEO | LiTFSI | CsPbI_3_ perovskite quantum dots | 0.14 | 0.57 |
| [S2] | PTFEP | LiFSI | N/A | 0.3 | 0.64 |
| [S3] | PTMG-HDI-BHDS | LiFSI | N/A | 0.24 | 0.81 |
| [S4] | PRX | LiNO3 | DMSO Hexamethylene diisocyanate  Methylene diphenyl 4,4'-diisocyanate | 5.93 | 0.71 |
| [S5] | PEO | LiTFSI | GDC LSGM | 0.19 | 0.26 |
| [S6] | PBO/PEO | LiTFSI | N/A | 0.28 | N/A |
| [S7] | PS-PMA | LiClO_4_ | N/A | 1.2 | N/A |
| [S8] | PI-b-PS-b-PEO | LiTFSI | THF | 2.2 | 0.7 |
| [S9] | PGTE | LiTFSI | Succinonitrile | 0.416 | N/A |
| [S10] | PVEC | LiTFSI | AIBN, Glass fiber | 2.1 | 0.4 |
| [S11] | MEMA | LiTFSI LiDFOB | SN FEC AIBN LLZT | 1.17 | 0.63 |
| [S12] | PEO | LiTFSI | ADCN | 0.144 | 0.38 |
| [S13] | PVDF-HFP | LiPF_6_ LiNO_3_ | FEC  THF DMC | 0.339 | 0.56 |
| [S14] | PSi-S-CN | LiTFSI | DME | 0.64 | 0.53 |
| [S15] | PVDF-HFP | LiTFSI | [BMIM]BF4 PC EC Al2O3 ACN | 5.26 | 0.23 |
| [S16] | PVDF ETT DODT | LiFSI LiDFOB LiPF_6_ | FEC FEMC EC DMPA | 4.41 | 0.51 |
| [S17] | DOL PS | LiPF_6_ LiFSI LiTFSI | DME | 2.22 | 0.88 |
| This work | PVA | LiClO_4_ | HT | 3.2 | 0.47 |

**Table S2** Activation energy of various previous studies

| Ref. | Polymers | Salts & additives | Redox material | Activation energy (eV) |
| --- | --- | --- | --- | --- |
| [S18] | PVdF-HFP | BMPTFSI SN  HQ | HQ | 0.181 |
| [S19] | PVA  PVP | TMAI KI | I | 0.13 |
| [S20] | PVdF-HFP | EMITf Al(Tf)_3_ | Al | 0.18 |
| [S21] | DGEBA PVdF-HFP | [DMIM]Br | Br | 0.29 |
| [S22] | PVdF-HFP | EMITFSI | N/A | 0.21 |
|  |  | EMIT-Br | Br | 0.22 |
|  |  | EMIT-I | I | 0.2 |
| This work | PVA | LiClO_4_ HT | HT | 0.13 |

**Table S3** Comparison of previous studies on similar VG@CNTF and electrolyte system.

|  | Cell Type | Electrode materials | Electrolytes | Energy density  (at power density) | Bending stability  (%/cycles number) | Cycle life  (%/cycles number) |
| --- | --- | --- | --- | --- | --- | --- |
| [S23] | FSS | 3D CNT fiber | H_2_SO_4_/PVA | 4.37 Wh kg^-1^  (50.38 W kg^-1^) | 90%  /1,000 | 74%  /2,000 |
| [S24] | FSS | MnO_2_@CNTF | LiCl/PVA | 4.82 Wh kg^-1^  (110 W kg^-1^) | 90%  /500 | 86%  /5,000 |
| [S25] | FSS | PANI@3D-CNF | H_3_PO_4_/PVA | 11.6 Wh kg^-1^  (24.5 W kg^-1^) | 100%  /20 | 74.2%  /3,000 |
| [S26] | FSS | N-doped G/CNT@CF | H_2_SO_4_/PVA | 11.1 Wh kg^-1^  (110 W kg^-1^) | N/A | 91%  /10,000 |
| [S27] | FSS | N-doped G/CNT fiber | H_3_PO_4_/PVA | 10.7 Wh kg^-1^  (3.1 kW kg^-1^) | 97%  /1,000 | 93%  /10,000 |
| [S28] | FSS | CNT@RF | H_3_PO_4_/PVA | 0.515 Wh kg^-1^  (421 W kg^-1^) | N/A | 90%  /1,000 |
| [S29] | FSS | PANI@CNTF | H_2_SO_4_/PVA | 7.3 Wh kg^-1^  (390.3 W kg^-1^) | 80%  /20,000 | 81%  /10,000 |
| [S30] | FSS | MoS_2_/r-GO/CNT fiber | H_2_SO_4_/PVA | 26.4 Wh kg^-1^  (4 kW kg^-1^) | 100%  /1,000 | 85%  /5,000 |
| [S31] | FSS | GF | H_2_SO_4_/PVA | 5.76 Wh kg^-1^  (47.3 W kg^-1^) | 91%  /1,000 | 92  /1,000 |
| [S32] | FSS | CNTF | H_2_SO_4_/PVA | 11.22 Wh kg^-1^  (1.4 kW kg^-1^) | N/A | 94.85%  /11,500 |
| [S33] | TBS | G/MoS_2_@GF | H_2_SO_4_/PVA | 10.42 Wh kg^-1^ (115.82 W kg^-1^) | 91%  /1,000 | 90%  /1,000 |
| [S34] | TBS | N-doped Carbon@r-GO@CNT | H_2_SO_4_/PVA | 19.4 Wh kg^-1^  (69.5 W kg^-1^) | 87%  /3,000 | 89%  /3,000 |
| [S35] | FTS | 3D Graphene | KOH | 8.65 Wh kg^-1^  (125 W kg^-1^) | N/A | 80%  /20,000 |
| [S36] | FTS | N-doped CNT/r-GO | KOH | 10 Wh kg^-1^ (1.4 kW kg^—1^) | N/A | 90.7%  /5,000 |
| [S37] | FTS | r-GO/CNT | H2SO4 | 12.8 Wh kg^-1^  (142.9 W kg^-1^) | N/A | 97.7%  /10,000 |
| Our work | FSS | VG@CNTF | HT/LiClO_4_  /PVA | 25.4 Wh kg^-1^  (25 kW kg^-1^)  17.1 Wh kg^-1^  (97 kW kg^-1^) | 91%  /8,000 | 83%  /10,000 |

*FSS: Fiber-shaped supercapacitor, TBS: Textile-based supercapacitor, FTS: Film-type supercapacitor

**Supplementary Reference**

[S1] H. Zhang, Wang, Y., Huang, J., Li, W., Zeng, X. et al. Low-Enthalpy and High-Entropy Polymer Electrolytes for Li-Metal Battery. Energy Environ. Mater. **7**, e12514 (2024). https://doi.org:https://doi.org/10.1002/eem2.12514

[S2] W. Zhang, Koverga, V., Liu, S., Zhou, J., Wang, J. et al. Single-phase local-high-concentration solid polymer electrolytes for lithium-metal batteries. Nat. Energy **9**, 386-400 (2024). https://doi.org:10.1038/s41560-023-01443-0

[S3] F. Pei, Wu, L., Zhang, Y., Liao, Y., Kang, Q. et al. Interfacial self-healing polymer electrolytes for long-cycle solid-state lithium-sulfur batteries. Nat. Commun. **15**, 351 (2024). https://doi.org:10.1038/s41467-023-43467-w

[S4] J. Seo, Lee, G.-H., Hur, J., Sung, M.-C., Seo, J.-H. et al. Mechanically Interlocked Polymer Electrolyte with Built-In Fast Molecular Shuttles for All-Solid-State Lithium Batteries. Adv. Energy Mater. **11**, 2102583 (2021). https://doi.org:https://doi.org/10.1002/aenm.202102583

[S5] N. Wu, Chien, P.-H., Qian, Y., Li, Y., Xu, H. et al. Enhanced Surface Interactions Enable Fast Li+ Conduction in Oxide/Polymer Composite Electrolyte. Angew. Chem.-Int. Edit. **59**, 4131-4137 (2020). https://doi.org:https://doi.org/10.1002/anie.201914478

[S6] A. Du, Lu, H., Liu, S., Chen, S., Chen, Z. et al. Breaking the Trade-Off between Ionic Conductivity and Mechanical Strength in Solid Polymer Electrolytes for High-Performance Solid Lithium Batteries. Adv. Energy Mater. **14**, 2400808 (2024). https://doi.org:https://doi.org/10.1002/aenm.202400808

[S7] Z. Zheng, Gao, X., Luo, Y. and Zhu, S. Employing Gradient Copolymer To Achieve Gel Polymer Electrolytes with High Ionic Conductivity. Macromolecules **49**, 2179-2188 (2016). https://doi.org:10.1021/acs.macromol.6b00021

[S8] A. Pelz, Dörr, T. S., Zhang, P., de Oliveira, P. W., Winter, M. et al. Self-Assembled Block Copolymer Electrolytes: Enabling Superior Ambient Cationic Conductivity and Electrochemical Stability. Chemistry of Materials **31**, 277-285 (2019). https://doi.org:10.1021/acs.chemmater.8b04686

[S9] Y. Ma, Sun, Q., Wang, S., Zhou, Y., Song, D. et al. Li salt initiated in-situ polymerized solid polymer electrolyte: new insights via in-situ electrochemical impedance spectroscopy. Chem. Eng. J. **429**, 132483 (2022). https://doi.org:https://doi.org/10.1016/j.cej.2021.132483

[S10]Z. Lin, Guo, X., Wang, Z., Wang, B., He, S. et al. A wide-temperature superior ionic conductive polymer electrolyte for lithium metal battery. Nano Energy **73**, 104786 (2020). https://doi.org:https://doi.org/10.1016/j.nanoen.2020.104786

[S11]A.-G. Nguyen, Lee, M.-H., Kim, J. and Park, C.-J. Construction of a High-Performance Composite Solid Electrolyte Through In-Situ Polymerization within a Self-Supported Porous Garnet Framework. Nano-Micro Lett. **16**, 83 (2024). https://doi.org:10.1007/s40820-023-01294-0

[S12]Y. Dai, Zhuang, M., Deng, Y.-X., Liao, Y., Gu, J. et al. Stable Cycling of All-Solid-State Lithium Batteries Enabled by Cyano-Molecular Diamond Improved Polymer Electrolytes. Nano-Micro Lett. **16**, 217 (2024). https://doi.org:10.1007/s40820-024-01415-3

[S13]H. Duan, You, Y., Wang, G., Ou, X., Wen, J. et al. Lithium-Ion Charged Polymer Channels Flattening Lithium Metal Anode. Nano-Micro Lett. **16**, 78 (2024). https://doi.org:10.1007/s40820-023-01300-5

[S14]C. Fu, Iacob, M., Sheima, Y., Battaglia, C., Duchêne, L. et al. A highly elastic polysiloxane-based polymer electrolyte for all-solid-state lithium metal batteries. J. Mater. Chem. A **9**, 11794-11801 (2021). https://doi.org:10.1039/D1TA02689E

[S15]K. Huang, Wang, Y., Mi, H., Ma, D., Yong, B. et al. [BMIM]BF4-modified PVDF-HFP composite polymer electrolyte for high-performance solid-state lithium metal battery. J. Mater. Chem. A **8**, 20593-20603 (2020). https://doi.org:10.1039/D0TA08169H

[S16]K. Deng, Xu, Z., Zhou, S., Zhao, Z., Zeng, K. et al. Nonflammable highly-fluorinated polymer electrolytes with enhanced interfacial compatibility for dendrite-free lithium metal batteries. J. Power Sources **510**, 230411 (2021). https://doi.org:https://doi.org/10.1016/j.jpowsour.2021.230411

[S17]K. Mu, Wang, D., Dong, W., Liu, Q., Song, Z. et al. Hybrid Crosslinked Solid Polymer Electrolyte via In-Situ Solidification Enables High-Performance Solid-State Lithium Metal Batteries. Adv. Mater. **35**, 2304686 (2023). https://doi.org:https://doi.org/10.1002/adma.202304686

[S18]N. Yadav, Yadav, N. and Hashmi, S. A. High-Energy-Density Carbon Supercapacitors Incorporating a Plastic-Crystal-Based Nonaqueous Redox-Active Gel Polymer Electrolyte. ACS Appl. Energ. Mater. **4**, 6635-6649 (2021). https://doi.org:10.1021/acsaem.1c00703

[S19]M. F. Aziz, Azam, M. A., Yusuf, S. N. F., Buraidah, M. H. and Arof, A. K. Influence of potassium iodide in polyvinyl alcohol-based gel polymer electrolyte for efficiency enhancement of dye-sensitized solar cells. J. Poly. Res. **29**, 455 (2022). https://doi.org/10.1007/s10965-022-03305-x

[S20]J. Liu, Khanam, Z., Ahmed, S., Wang, H., Wang, T. et al. A study of low-temperature solid-state supercapacitors based on Al-ion conducting polymer electrolyte and graphene electrodes. J. Power Sources **488**, 229461 (2021). https://doi.org:https://doi.org/10.1016/j.jpowsour.2021.229461

[S21]L. C. Oliveira da Silva and Soares, B. G. New all solid-state polymer electrolyte based on epoxy resin and ionic liquid for high temperature applications. J. Appl. Polym. Sci. **135**, 45838 (2018). https://doi.org:https://doi.org/10.1002/app.45838

[S22]B. Asbani, Douard, C., Brousse, T. and Le Bideau, J. High temperature solid-state supercapacitor designed with ionogel electrolyte. Energy Storage Mater. **21**, 439-445 (2019). https://doi.org:https://doi.org/10.1016/j.ensm.2019.06.004

[S23]Y. Li, Kang, Z., Yan, X., Cao, S., Li, M. et al. A facile method for the preparation of three-dimensional CNT sponge and a nanoscale engineering design for high performance fiber-shaped asymmetric supercapacitors. Journal of Materials Chemistry A **5**, 22559-22567 (2017). https://doi.org:10.1039/C7TA06722D

[S24]C. Ren, Yan, Y., Sun, B., Gu, B. and Chou, T.-W. Wet-spinning assembly and in situ electrodeposition of carbon nanotube-based composite fibers for high energy density wire-shaped asymmetric supercapacitor. Journal of Colloid and Interface Science **569**, 298-306 (2020). https://doi.org:https://doi.org/10.1016/j.jcis.2020.02.092

[S25]D. Yang, Ni, W., Cheng, J., Wang, Z., Li, C. et al. Omnidirectional porous fiber scrolls of polyaniline nanopillars array-N-doped carbon nanofibers for fiber-shaped supercapacitors. Materials Today Energy **5**, 196-204 (2017). https://doi.org:https://doi.org/10.1016/j.mtener.2017.06.011

[S26]G. Zhou, Kim, N.-R., Chun, S.-E., Lee, W., Um, M.-K. et al. Highly porous and easy shapeable poly-dopamine derived graphene-coated single walled carbon nanotube aerogels for stretchable wire-type supercapacitors. Carbon **130**, 137-144 (2018). https://doi.org:https://doi.org/10.1016/j.carbon.2017.12.123

[S27]D. Yu, Goh, K., Wang, H., Wei, L., Jiang, W. et al. Scalable synthesis of hierarchically structured carbon nanotube–graphene fibres for capacitive energy storage. Nature Nanotechnology **9**, 555-562 (2014). https://doi.org:10.1038/nnano.2014.93

[S28]Z. Yang, Deng, J., Chen, X., Ren, J. and Peng, H. A Highly Stretchable, Fiber-Shaped Supercapacitor. Angewandte Chemie International Edition **52**, 13453-13457 (2013). https://doi.org:https://doi.org/10.1002/anie.201307619

[S29]S. Lee, Kim, J.-G., Yu, H., Lee, D.-M., Hong, S. et al. Flexible supercapacitor with superior length and volumetric capacitance enabled by a single strand of ultra-thick carbon nanotube fiber. Chemical Engineering Journal **453**, 139974 (2023). https://doi.org:https://doi.org/10.1016/j.cej.2022.139974

[S30]X. Jian, Li, H., Li, H., Li, Y. and Shang, Y. Flexible and freestanding MoS2/rGO/CNT hybrid fibers for high-capacity all-solid supercapacitors. Carbon **172**, 132-137 (2021). https://doi.org:https://doi.org/10.1016/j.carbon.2020.09.095

[S31]S. Chen, Ma, W., Cheng, Y., Weng, Z., Sun, B. et al. Scalable non-liquid-crystal spinning of locally aligned graphene fibers for high-performance wearable supercapacitors. Nano Energy **15**, 642-653 (2015). https://doi.org:https://doi.org/10.1016/j.nanoen.2015.05.004

[S32]Y. Zhou, Cai, Y., Tu, T., Zhang, S., Li, T. et al. Expanded Carbon Nanotube Fiber at the Liquid–Air Interface for High-Performance Fiber-Based Supercapacitors and Electrochemical Sensors. ACS Applied Materials & Interfaces **15**, 41839-41849 (2023). https://doi.org:10.1021/acsami.3c06815

[S33]M. Dulal, Islam, M. R., Maiti, S., Islam, M. H., Ali, I. et al. Smart and Multifunctional Fiber-Reinforced Composites of 2D Heterostructure-Based Textiles. Advanced Functional Materials **33**, 2305901 (2023). https://doi.org:https://doi.org/10.1002/adfm.202305901

[S34]N. Lingappan, Lim, S., Lee, G.-H., Luan, V. H., Jeon, I. et al. Carbon triple-junction nanostructures for solid-state stretchable and flexible fabric/yarn-type symmetric supercapacitors. Journal of Energy Storage **83**, 110733 (2024). https://doi.org:https://doi.org/10.1016/j.est.2024.110733

[S35]V. H. Pham, Wang, C., Gao, Y., Weidman, J., Kim, K.-J. et al. Synthesis of Microscopic 3D Graphene for High-Performance Supercapacitors with Ultra-High Areal Capacitance. Small Methods **8**, 2301426 (2024). https://doi.org:https://doi.org/10.1002/smtd.202301426

[S36]X. Li, Xu, Y., Hu, G., Luo, Z., Xu, D. et al. Self-assembled formation of conjugated 3D reduced graphene oxide-wrapped helical CNTs nanostructure and nitrogen-doped using photochemical doping for high-performance supercapacitor electrodes. Electrochimica Acta **280**, 33-40 (2018). https://doi.org:https://doi.org/10.1016/j.electacta.2018.05.106

[S37]L. Yao, Zhou, C., Hu, N., Hu, J., Hong, M. et al. Flexible graphene/carbon nanotube hybrid papers chemical-reduction-tailored by gallic acid for high-performance electrochemical capacitive energy storages. Applied Surface Science **435**, 699-707 (2018). https://doi.org:https://doi.org/10.1016/j.apsusc.2017.11.163
